# Serum Proteomics Provides Novel Biomarkers of Inflammation, Tissue Injury, and Therapeutic Response in Experimental Chagas Disease

**DOI:** 10.3390/microorganisms14030588

**Published:** 2026-03-05

**Authors:** Eloan Mendes Vieira, Camilo Elber Vital, Paula Melo de Abreu Vieira, Lorena Cera Bandeira, Luciana da Fonseca Medeiros, Nívia Carolina Nogueira Paiva, William de Castro Borges, Cláudia Martins Carneiro

**Affiliations:** 1 Laboratory of Immunopathology, Nucleus of Biological Sciences Research, Institute of Exact and Biological Sciences, Federal University of Ouro Preto, Ouro Preto 35400-000, Brazil; eloanmv@gmail.com (E.M.V.); lorena.bandeira@ufop.edu.br (L.C.B.); luciana.medeiros@ufop.edu.br (L.d.F.M.); niviacarolinanp@ufop.edu.br (N.C.N.P.); 2 Enzymology and Proteomics Laboratory, Department of Biological Sciences, Nucleus of Biological Sciences Research, Institute of Exact and Biological Sciences, Federal University of Ouro Preto, Ouro Preto 35400-000, Brazil; camilo.vital@ufop.edu.br (C.E.V.); wborges@ufop.edu.br (W.d.C.B.); 3 Laboratory of Morphopathology, Department of Biological Sciences, Nucleus of Biological Sciences Research, Institute of Exact and Biological Sciences, Federal University of Ouro Preto, Ouro Preto 35400-000, Brazil; paula@ufop.edu.br

**Keywords:** Chagas disease, *Trypanosoma cruzi*, serum proteomics, benznidazole response, inflammatory biomarkers, drug resistance

## Abstract

Chagas disease remains a major public health challenge and still lacks reliable serum biomarkers capable of accurately reflecting disease progression and therapeutic response. Here, we performed a quantitative label-free serum proteomic analysis in a murine model infected with two *Trypanosoma cruzi* strains exhibiting contrasting sensitivity to benznidazole (Be-78, sensitive; VL-10, resistant), evaluated during both acute and chronic phases, in the presence or absence of treatment. Distinct proteomic signatures were observed across strains, infection stages, and experimental groups, involving pathways related to complement activation, inflammatory responses, immunoglobulins, energy metabolism, and tissue remodeling. Markers of cellular injury, including LDH-A, C1q, and C6, remained predominantly elevated in mice infected with the benznidazole-resistant strain, whereas animals infected with Be-78 showed substantial proteomic normalization following treatment. In addition, structural proteins such as dystrophin, nebulin, alpha-adducin, and myosin XVIIIb clearly distinguished strain-dependent profiles of disease aggressiveness and tissue damage. Integrated analyses revealed that benznidazole efficacy is strongly influenced by the biological characteristics of the infecting strain and is directly mirrored in the serum proteome. Collectively, these findings identify promising serum biomarkers of tissue injury and therapeutic response and underscore the importance of parasite genetic variability in disease monitoring and in the development of improved diagnostic and therapeutic strategies.

## 1. Introduction

Chagas disease, caused by the protozoan *Trypanosoma cruzi*, remains a serious public health problem in Latin America and in several non-endemic countries, driven by population mobility. It is estimated that 6–7 million people are infected worldwide, of whom 30–40% progress to chronic cardiac or digestive forms characterized by persistent inflammation and progressive tissue damage [1,2]. Although diagnostic advances have been achieved, robust serum biomarkers capable of predicting clinical progression or accurately monitoring therapeutic response—particularly during the chronic phase—are still lacking [3,4,5,6].

The genetic variability of *T. cruzi* is one of the main determinants of the clinical heterogeneity observed, influencing virulence, tissue tropism, inflammatory responses, and therapeutic efficacy. The different discrete typing units (DTUs) exhibit distinct patterns of replication, immune evasion, and susceptibility to benznidazole (BNZ) [7,8]. Currently, etiological therapy relies mainly on BNZ, which is the most widely used and first-line drug in most endemic regions. In addition, nifurtimox (NFX) is also approved and employed in several countries, particularly in cases of BNZ intolerance or therapeutic failure, despite its higher toxicity and lower tolerability [1,3]. Although treatment is effective during the acute phase, its impact in the chronic phase remains limited, with inconsistent rates of parasitological cure and a lack of reliable markers for therapeutic evaluation [4].

Recent evidence shows that host–parasite interactions profoundly modulate pathways associated with the complement system, immunoglobulins, cytokines, oxidative stress, energy metabolism, and structural remodeling—processes closely linked to the development of Chagas cardiomyopathy [1,9,10]. Proteomic analysis has emerged as a powerful tool for unraveling molecular pathways associated with disease severity, inflammatory intensity, and treatment outcomes. However, most studies have focused on patients, isolated tissues, or the parasite secretome, and there is a shortage of integrative approaches that simultaneously evaluate: (i) distinct strains with contrasting profiles; (ii) acute and chronic phases; and (iii) the direct effect of the drug.

Thus, a critical gap remains in the literature: the absence of serum proteomic studies capable of characterizing how *T. cruzi* genetic variability and pharmacological therapy remodel the seroproteome throughout infection. Such approaches are essential for identifying robust biomarkers of inflammation, tissue injury, and therapeutic response, as well as for improving the understanding of BNZ resistance mechanisms.

In this context, the present study applies quantitative label-free proteomics to comprehensively characterize the serum proteome of mice infected with two *T. cruzi* strains exhibiting contrasting sensitivity to benznidazole (Be-78, sensitive; VL-10, resistant). Animals were evaluated during both acute and chronic phases, in the presence or absence of treatment. The primary aim was to identify strain- and stage-specific proteomic signatures associated with inflammation, tissue injury, and therapeutic response, as well as to propose candidate serum biomarkers for disease monitoring.

## 2. Materials and Methods

### 2.1. Animals, T. cruzi Strains, and Experimental Infection

All procedures involving animals were conducted in accordance with the guidelines of the National Council for the Control of Animal Experimentation (CONCEA) and international standards of animal welfare. The study was approved by the Animal Ethics Committee of the Federal University of Ouro Preto (CEUA/UFOP), protocol 4055030718.

**Figure 1:**
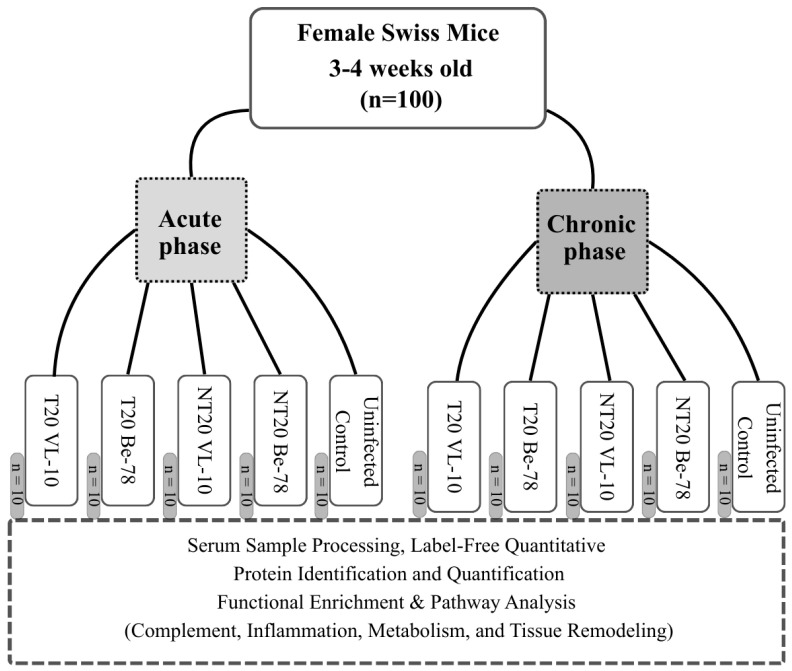
The study design. Female Swiss mice were infected with benznidazole-sensitive (Be-78) or resistant (VL-10) *Trypanosoma cruzi* strains and evaluated during acute and chronic infection, in the presence (T20) or absence of treatment (NT20) (*n* = 10 per group). Serum samples were analyzed using label-free quantitative proteomics to identify differentially expressed proteins and enriched biological pathways. Comparative analyses enabled the identification of proteomic signatures associated with strain, infection stage, and therapeutic response, supporting the discovery of candidate biomarkers.

A total of 100 female Swiss mice, 3–4 weeks old (≈22 g), were used (Figure 1). Animals were maintained under controlled conditions (23 ± 2 °C, 12 h light/dark cycle, forced ventilation) with food and water provided ad libitum. They were evenly distributed between the acute (*n* = 50) and chronic (*n* = 50) phases of infection. For each infection phase and for each *T. cruzi* strain, animals were allocated into three experimental groups: uninfected control (CN; *n* = 10), infected and untreated (NT20; *n* = 10), and infected and treated with BNZ for 20 days (T20; *n* = 10). This experimental design was applied independently for the Be-78 (BNZ-sensitive) and VL-10 (BNZ-resistant) strains.

Mice were infected with blood-derived trypomastigote forms obtained from mice previously infected with the Be-78 (BNZ-sensitive) and VL-10 (BNZ-resistant) *T. cruzi* strains. Both strains belong to the TcII discrete typing unit (DTU) and have been extensively characterized regarding their biological behavior and differential susceptibility to BNZ in experimental murine models. The Be-78 strain is considered susceptible, whereas the VL-10 strain exhibits a resistant phenotype, as previously described [8,11].

The strains were maintained by serial passages in mice to ensure phenotypic stability. Inoculation was performed by intraperitoneal injection of 5 × 10^3^ parasites diluted in 1× PBS. Infection was confirmed on day 7 post-inoculation by optical microscopy using the fresh-smear technique [8,12].

### 2.2. Pharmacological Treatment

BNZ (Pharmaceutical Laboratory of Pernambuco (Lafepe, Brazil)) was prepared in a 0.5% methylcellulose suspension (Sigma-Aldrich (St. Louis, MO, USA)). After infection was confirmed, animals in the T20 group received BNZ via oral gavage at 100 mg/kg/day for 20 consecutive days. Acute phase: treatment initiated on day 7 post-infection. Chronic phase: treatment initiated on day 180 post-infection [8,11].

### 2.3. Euthanasia and Sample Collection

At the end of the experimental periods, animals were anesthetized with ketamine (100 mg/kg) and xylazine (9 mg/kg) and euthanized following CONCEA-approved procedures. Blood was collected by cardiac puncture, centrifuged at 12,000 rpm for 12 min, and the separated serum was stored at −80 °C until further analysis [8].

### 2.4. Protein Quantification and Sample Preparation for Proteomics

Total protein concentration was determined using the BCA method (Pierce™ BCA Protein Assay Kit (Thermo Fisher Scientific, Waltham, MA, USA)). Proteomic analyses were performed using individual serum samples, without sample pooling. For each experimental group, three serum samples were randomly selected from the ten animals available for proteomic analyses. For each sample, 50 µg of serum protein was subjected to tryptic digestion. The protocol included:

Denaturation with RapiGest™ (1%) at 80 °C for 10 min;

Reduction with DTT (9.2 mg/mL) at 60 °C for 10 min;

Alkylation with IAA (33 mg/mL) in the dark for 40 min;

Digestion with trypsin (50:1 ratio) for 18 h at 37 °C;

Surfactant degradation with TFA (1%) at 37 °C for 40 min;

Centrifugation at 20,000× *g* for 20 min, followed by collection of the peptide-containing supernatant [13].

### 2.5. Liquid Chromatography and Mass Spectrometry (UHPLC-MS/MS)

Peptides were analyzed in a Dionex UltiMate 3000 UHPLC system coupled to a Q Exactive mass spectrometer (Thermo Fisher Scientific, Waltham, MA, USA). Initial purification was performed using an Acclaim PepMap100 C18 NanoTrap cartridge (Thermo Fisher Scientific, Waltham, MA, USA), followed by separation on an Easy-Spray C18 analytical column (75 µm × 10 cm).

Elution was carried out using an acetonitrile gradient (3.8–40%) over 110 min at a flow rate of 0.3 µL/min. Ionization was performed by nanospray (3.45 kV spray voltage; capillary temperature 250 °C).

Data acquisition followed a Data-Dependent Acquisition (DDA) Top12 strategy with the following settings: resolution of 70,000 (MS1) and 35,000 (MS2); *m*/*z* range 300–2000; dynamic exclusion of 40 ms; charge states +2 to +4 [13].

### 2.6. Protein Identification and Quantification

Raw files were processed using PEAKS Studio v8.5 (Bioinformatics Solutions Inc., Waterloo, ON, Canada). Parameters included: tryptic digestion; up to 2 missed cleavages; carbamidomethylation (Cys) as a fixed modification; oxidation (Met) as a variable modification; up to 3 modifications per peptide; mass tolerance of 10 ppm (MS1) and 0.1 Da (MS2); FDR ≤ 1%; statistical significance *p* ≤ 0.05; and protein acceptance with ≥1 unique peptide [13].

Identifications were performed against the UniProt *Mus musculus* (C57BL/6J) database (55,311 sequences; accessed on 2 December 2022).

### 2.7. Statistical and Bioinformatic Analyses

Normalization of intensities, relative (label-free) quantification, and determination of differentially abundant proteins were performed directly in PEAKS Studio. Pathway analyses and prediction were conducted using the PANTHER v18.0 software with default parameters.

### 2.8. Data Availability

Mass spectrometry proteomics data were deposited in the ProteomeXchange Consortium via the PRIDE partner repository under dataset identifier PXD056503. Dataset name: Proteomic approaches using UHPLC-MS/MS in serum from mice infected with * T. cruzi*.

## 3. Results

### 3.1. Global Serum Proteome Profile and Dataset Quality

The UHPLC-MS/MS approach enabled the identification of a robust and reproducible set of serum proteins across all experimental conditions. In the acute phase, 517 proteins were identified in the Be-78 strain and 506 in the VL-10 strain (Table S1); in the chronic phase, 380 and 422 proteins, respectively (Table S2). These numbers reflect good analytical depth, appropriate coverage of the serum proteome, and technical consistency among samples and experimental groups. Complete spectral acquisition indicators—including the number of MS/MS scans, PSMs, identified peptides, and the distribution of unique peptides per protein—are detailed in Table 1 and Table 2.

**Figure 2:**
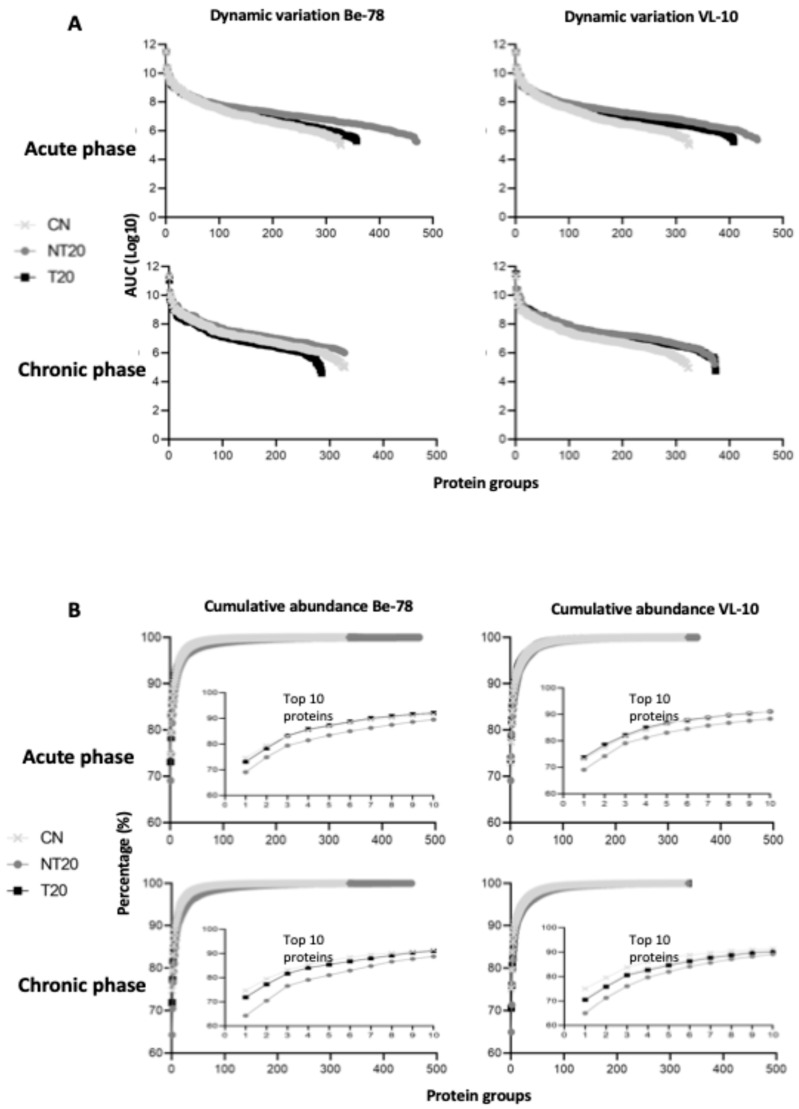
(**A**) Dynamic variation of proteins on a logarithmic scale (log_10_), demonstrating protein detection across approximately five orders of magnitude using the UHPLC-MS/MS platform. (**B**) cumulative protein abundance in the serum proteome of infections caused by the Be-78 and VL-10 strains during the acute and chronic phases, including the CN, NT20, and T20 groups. The inset plots highlight the relative contribution of the 10 most abundant proteins under each condition.

The global abundance distribution revealed a dynamic range of approximately five orders of magnitude (Figure 2), typical of shotgun analyses in non-depleted serum. The top 10 most abundant proteins in the samples were examined (Table S3), among which albumin accounted for 65–75% of total abundance under all conditions—a pattern widely documented in similar studies, as demonstrated by [13,14]. Despite this predominance, the method enabled the detection of a large number of biologically relevant proteins, highlighting the strong performance of the analytical platform.

In the infected and untreated groups (NT20), a proportional reduction in albumin was observed, accompanied by a marked increase in proteins related to inflammatory responses, iron/heme metabolism, the complement system, and immunoglobulins—a pattern consistent with the pathophysiology of *T. cruzi* infection [1]. Among these proteins, serotransferrin stands out for its essential role in limiting iron availability to pathogens [15], as well as hemopexin, a key component in the sequestration of free heme and mitigation of oxidative damage [16]. The elevation of these proteins reinforces the profound disruption of iron/heme metabolism during infection.

Pregnancy zone protein (PZP) also showed a pronounced increase, particularly in VL-10 infection. Previous studies have reported its elevation during the acute phase of experimental Chagas disease, and its participation in systemic inflammatory processes [17,18], supporting its relevance as a potential inflammation marker.

Another prominent component was Complement C3, identified among the most abundant proteins in infected groups. In addition to its central role in innate immunity, C3 also influences T-cell modulation during chronic infection, underscoring its involvement in the systemic response observed here. The concurrent elevation of C3 and multiple immunoglobulins is consistent with the persistent immune activation characteristic of *T. cruzi* infection [19].

Apolipoprotein A-I (ApoA1) showed a marked reduction in NT20 groups, with partial or complete normalization following BNZ treatment, especially in Be-78. This behavior aligns with the role of ApoA1 in lipid metabolism and innate immunity. Studies have shown that cruzipain, the major *T. cruzi* protease, can cleave ApoA1 [20], contributing to its reduction during infection. Moreover, ref. [21] identified ApoA1 as a candidate biomarker of therapeutic response, reinforcing the consistency of the patterns observed in this study.

In the chronic phase, an increase in alpha-1B-glycoprotein (A1BG) was observed. Although its function remains partially understood, it has been associated with modulation of inflammatory responses through interaction with CRISP3 [22]. Its elevation may reflect late immunological adaptation mechanisms in the context of persistent inflammation.

Taken together, these findings demonstrate that although albumin is the dominant protein in the serum proteome, infection drives profound remodeling of serum proteins, particularly in NT20 groups. Pathways associated with innate immunity, oxidative stress, iron/heme metabolism, systemic inflammation, and complement activation were extensively modulated. BNZ treatment reversed part of these alterations, more markedly in the Be-78 strain, whereas VL-10 exhibited more modest modulation, consistent with its lower drug sensitivity. These results reinforce that intrinsic differences among *T. cruzi* strains and the degree of parasite control directly influence the seroproteome, as discussed by [1,14].

### 3.2. Distinct Sets of Proteins Exclusively Detected Across Groups

**Figure 3:**
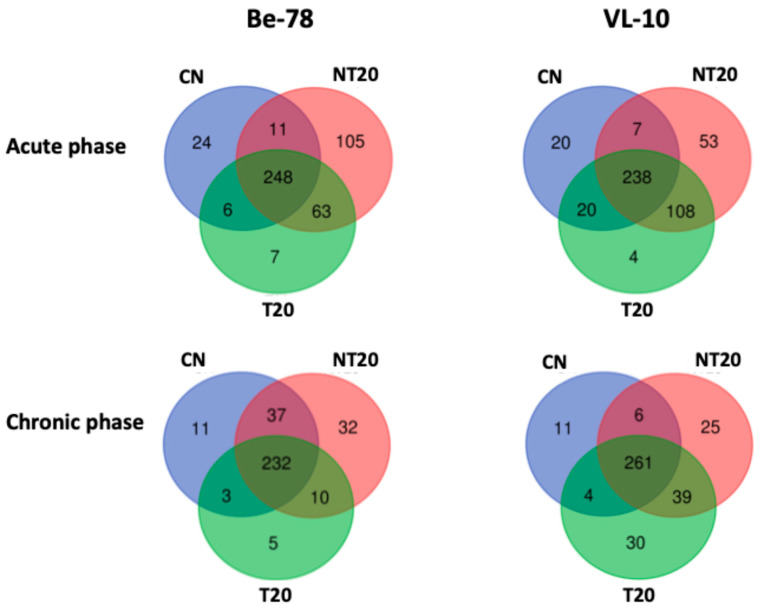
Distribution of exclusive and shared serum proteins among CN, NT20, and T20 groups in infections caused by the Be-78 and VL-10 strains during the acute and chronic phases.

Analysis of the sets of proteins exclusively detected under each experimental *T. cruzi* condition revealed distinct patterns between the acute and chronic phases and between the Be-78 and VL-10 strains (Figure 3; Table S4). These profiles reflect differences in the degree of inflammatory activation, immune modulation, and tissue remodeling—phenomena widely associated with the pathogenesis of infection [2,19].

In the Be-78 acute phase, the NT20 group showed the highest number of exclusive proteins (105), composed mainly of immunoglobulins and components of the classical complement pathway, consistent with the intense activation of the humoral immune response characteristic of the acute phase [2,19]. Among the exclusive proteins with the greatest functional relevance were: Chymotrypsin-like elastase 2α, involved in metabolic mechanisms and inflammatory responses; Complement C2, a central component of the classical complement pathway; and Clusterin (fragment), associated with inflammation, apoptosis, and tissue remodeling processes.

The T20 group infected with the Be-78 strain presented only 7 exclusive proteins, suggesting reduced inflammatory activity and partial normalization of the seroproteome after treatment, in agreement with the expected behavior for a drug-sensitive strain [21,23].

In the chronic phase (Be-78 infection), 32 exclusive proteins were identified in the NT20 group, including multiple immunoglobulins and complement regulators, indicating persistent immune activation even long after infection—a well-established phenomenon in chronic Chagas disease [2,10]. Notable proteins included: Tetranectin, associated with muscle regeneration and tissue remodeling; Complement factor H-related 4, a modulator of the alternative complement pathway frequently exploited by *T. cruzi* for immune evasion [19]. On the other hand, the T20 group presented only 5 exclusive proteins, reinforcing proteomic stabilization after treatment.

In the VL-10 strain acute phase, the NT20 group exhibited 53 exclusive proteins, including several IgG chains; Complement C6, a classical marker of terminal complement activation; and structural and cell-junction proteins such as Ezrin, Junction plakoglobin, and MACF1. This set indicates: strong activation of innate and adaptive immunity [19] and alterations in proteins essential for cardiac integrity, consistent with structural lesions described in Chagas cardiomyopathy [23].

The T20 group infected with the VL-10 strain showed only 4 exclusive proteins, suggesting a limited impact of treatment on seroproteome modulation in this resistant strain.

In the chronic phase of the infection with the VL-10 strain, the NT20 group displayed 25 exclusive proteins, including immunoglobulins, complement regulators, and structural proteins. Notably, Nebulin, associated with myofibril organization and muscle contractility, was identified—frequently linked to muscle damage in chronic cardiac disease [23].

Interestingly, the T20 group presented 30 exclusive proteins, a number higher than that observed for Be-78, including proteins related to muscle contractility, energy metabolism, and tissue remodeling. This pattern suggests that even after treatment, the VL-10 strain maintains a highly modulated seroproteome, possibly reflecting compensatory processes and post-infection adaptation mechanisms, consistent with observations in chronic models with low therapeutic responsiveness [24].

### 3.3. Differentially Abundant Proteins in the Be-78 Strain

**Figure 4:**
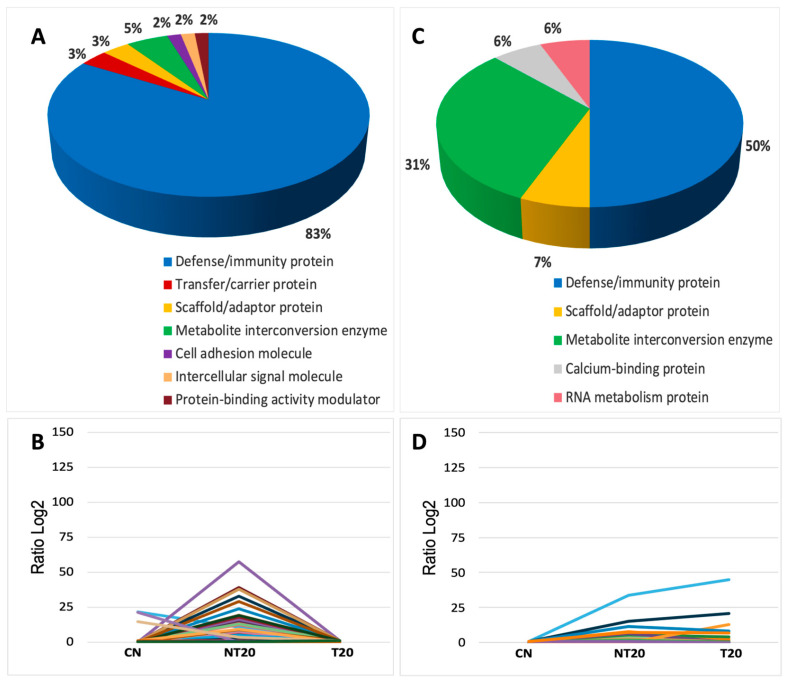
Analysis of differentially abundant serum proteins in the Be-78 strain during the acute and chronic phases of *T. cruzi* infection. (**A**) Functional classification of modulated proteins in the acute phase. (**B**) Abundance variation (Log_2_ ratio) of proteins in the acute phase. (**C**) Functional classification of modulated proteins in the chronic phase. (**D**) Abundance variation (Log_2_ ratio) of proteins in the chronic phase.

Quantitative analysis identified 67 differentially abundant proteins in the acute phase of infection with the Be-78 strain (Figure S1 and Figure 4; Table S5). The observed profile reflects an intense and highly modulated systemic inflammatory response, characterized by predominance of proteins related to complement activation, immunoglobulins, and mediators associated with immune activation.

The NT20 group showed marked increases in several components of innate and adaptive immunity. Among the most modulated proteins, C1qA and C1qB stood out (3–4-fold higher than CN), confirming strong activation of the classical complement pathway. This trend paralleled a robust expansion of immunoglobulins—more than 50 heavy-, kappa-, and lambda-chain immunoglobulins exhibited significant elevation, with log2 fold-changes exceeding 15–30 for many of them. This pattern indicates an exuberant humoral response, typical of the peak acute phase in drug-sensitive models.

Proteins involved in immune recognition and regulation, such as BPI-fold–containing family A2, CD5 antigen-like, and Cathepsin S, also showed marked increases, reflecting amplification of the inflammatory response and antigen-processing activity. In addition, proteins related to adhesion and cell recruitment, such as VCAM-1, were strongly elevated in the NT20 group, indicating endothelial activation consistent with the pathophysiology described for the acute phase of Chagas disease.

Interestingly, coagulation-associated proteins displayed behavior opposite to what is typically expected in intense inflammatory states. The Coagulation factor XIII A chain was completely suppressed in the NT20 group, suggesting impaired clot stability or excessive consumption during inflammation. Similarly, the metabolic marker L-lactate dehydrogenase A chain (LDH-A) showed a pronounced reduction in NT20 and complete absence in T20. This result may reflect negative metabolic regulation, increased systemic consumption, or reduced tissue release into serum, in contrast to classical patterns of cellular injury.

T20 produced strong normalization of the seroproteome. Most proteins elevated in NT20 returned to values near CN levels, including immunoglobulins, C1q, and inflammatory mediators. Proteins such as VCAM-1 and alpha-enolase decreased to levels below those of controls, indicating a potent anti-inflammatory effect and metabolic rebalancing induced by therapy. This set of findings clearly demonstrates the high therapeutic responsiveness of the Be-78 strain, consistent with its BNZ-susceptible phenotype.

In summary, the acute phase of Be-78 infection is marked by intense activation of complement and immunoglobulin pathways, accompanied by strong modulation of inflammatory mediators and unexpected alterations in metabolic and coagulation proteins. Treatment restored most modulated pathways, reinforcing the therapeutic efficacy of BNZ in this model.

In the Be-78 chronic phase of Be-78 infection, 22 differentially abundant proteins were identified (Table S6). Although fewer than in the acute phase, these markers reflect key chronic processes, including persistent immune activation, tissue remodeling, and metabolic modulation.

The NT20 group exhibited substantial increases in complement system components, particularly C1qA, which reached levels more than 30-fold higher than CN, suggesting sustained classical complement activation long after infection. Several heavy-, kappa-, and lambda-chain immunoglobulins—such as Ig HV11-1, HV1-39, HV1-80, kappa 4-51, and lambda C-2—also displayed marked elevations, indicating maintenance of humoral activity, a common pattern in chronic murine models with residual antigenemia.

Beyond immune processes, proteins related to tissue physiology and extracellular matrix organization remained modulated. EMILIN-2 showed evident increases in both NT20 and T20 groups, suggesting involvement in remodeling, tissue repair, and structural reorganization—hallmarks of chronic *T. cruzi*–induced cardiomyopathy. Similarly, Creatine kinase M-type, central to muscle energy metabolism, remained altered, reflecting possible cardiac metabolic stress.

Another notable protein was NEDD8 ultimate buster 1 (NUB1), which was significantly elevated in the treated group (T20). This pattern suggests that ubiquitin-like regulatory pathways play an important role during chronic infection, potentially associated with proteostasis control and post-treatment rebalancing. The presence of proteins such as Parvalbumin-α, involved in intracellular calcium homeostasis, reinforces the contribution of excitation–contraction mechanisms, frequently affected in cardiac dysfunction models.

T20 produced mixed outcomes: several inflammatory and immunological proteins were partially normalized, whereas others—such as C1qA, EMILIN-2, and some immunoglobulins—remained elevated. This partial modulation suggests that, although BNZ substantially reduces inflammation, certain chronic processes persist, reflecting an incomplete therapeutic response compared with the acute phase.

In summary, the chronic phase of Be-78 infection displays a more stable proteomic signature, yet one characterized by continued complement activation and humoral response, along with structural and metabolic alterations consistent with chronic cardiac remodeling. BNZ treatment reduces part of these changes but does not fully restore the seroproteome to baseline.

### 3.4. Differentially Abundant Proteins in the VL-10 Strain

**Figure 5:**
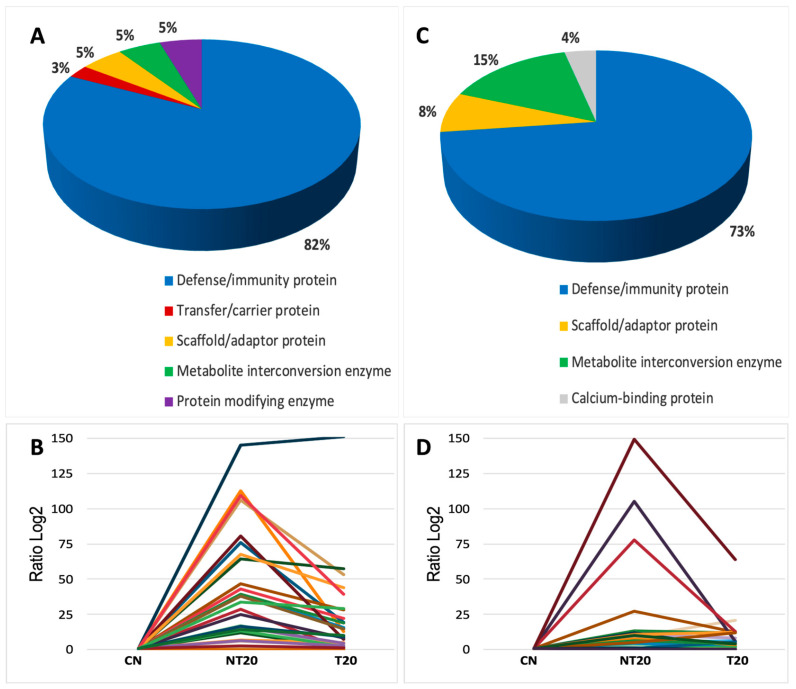
Analysis of differentially abundant serum proteins in the VL-10 strain during the acute and chronic phases of *T. cruzi* infection. (**A**) Functional classification of modulated proteins in the acute phase. (**B**) Abundance variation (Log_2_ ratio) of proteins in the acute phase. (**C**) Functional classification of modulated proteins in the chronic phase. (**D**) Abundance variation (Log_2_ ratio) of proteins in the chronic phase.

The acute phase of the VL-10 strain—recognized for its low sensitivity to BNZ—displayed a proteomic signature markedly distinct from that observed for the Be-78 strain. Differentially abundant proteins were identified that reflect an intense inflammatory response, greater tissue injury, and exacerbated activation of complement pathways, with only partial modulation following treatment (Figure S2 and Figure 5; Table S7).

The NT20 group exhibited a pronounced increase in classical markers of inflammation and cytotoxicity. Among these, the striking elevation of Complement C6 stood out, with abundance levels exceeding 100-fold relative to CN, demonstrating strong activation of the terminal complement pathway (membrane attack complex). This pattern is characteristic of amplified inflammatory processes and is commonly associated with increased tissue damage in resistant *T. cruzi* models.

Markers of immune recognition and inflammation, such as Cathepsin S and CD5 antigen-like, showed substantial increases, indicating heightened activation of lysosomal mechanisms, antigen processing, and immune regulation. In addition, several complement components—including C1qA and C1qB—were significantly elevated, reinforcing the immune hyperreactivity characteristic of the VL-10 strain during the acute phase.

The pattern of tissue injury was particularly evident in this strain. Structural proteins and proteins associated with cellular integrity appeared modulated, including fragments of cytoskeletal proteins and regulators of cellular dynamics. This pattern suggests a more severe structural compromise compared with the sensitive strain. The presence of albumin depletion and increased levels of low-molecular-weight inflammatory proteins also contributes to this scenario of greater pathogenic aggressiveness.

T20 induced only a partial reduction of most modulated proteins. Although consistent decreases were observed in C6, C1qA/B, and Cathepsin S, their levels remained far above those of CN, indicating that treatment was unable to fully reverse the inflammatory state. This behavior aligns with the natural BNZ-resistant phenotype of the VL-10 strain.

The set of differentially abundant immunoglobulins—although numerically smaller than in Be-78—showed moderate elevation in NT20, reinforcing the involvement of the humoral response, albeit with a less exuberant pattern. This may reflect intrinsic differences in inflammatory dynamics and antigenemia profiles between the two strains.

In summary, the acute phase of VL-10 infection is characterized by complement hyperactivation, robust markers of tissue damage, exacerbated inflammatory responses, and limited therapeutic modulation, forming a proteomic profile consistent with high virulence and low BNZ sensitivity. This contrasts with Be-78 highlights the importance of parasite genetic variability in shaping the seroproteome and determining therapeutic efficacy.

In the chronic phase of VL-10 infection, differentially abundant proteins revealed a persistent scenario of immune activation, tissue remodeling, and structural alterations, consistent with the resistant phenotype of this strain (Table S8). The observed pattern indicates that even after a long infection period, animals maintain a highly modulated seroproteome, suggesting residual inflammation, ongoing cardiac remodeling, and incomplete parasite control.

In the NT20 group, consistent increases were observed in several heavy-, kappa-, and lambda-chain immunoglobulins, reflecting maintenance of the humoral response typical of chronicity in resistant *T. cruzi* models. In addition, regulatory proteins of the Complement system components, including C1qA and fragments of alternative pathway proteins, remained elevated, indicating sustained immune activation.

A striking aspect of the chronic phase of VL-10 infection was the modulation of structural and myofibrillar proteins. Among these, Nebulin stood out as an essential protein for the organization of thin actin filaments and myofibrillar integrity. Its increase in the NT20 group suggests persistent muscle damage or activation of late regenerative mechanisms, phenomena already described in contexts of chronic cardiomyopathy. Other proteins involved in muscle architecture, energy metabolism, and contractility were also modulated, reinforcing the hypothesis of continuous tissue remodeling even at advanced stages of infection.

In the treated group (T20), partial reduction of several inflammatory and structural proteins was observed; however, the total number of differentially abundant proteins and the magnitude of their variation remained high when compared with the Be-78 strain. Notably, the VL-10 T20 group exhibited more exclusive proteins than chronic Be-78 itself, indicating that even after BNZ treatment, the seroproteome of these animals remains extensively altered. This behavior suggests that treatment was insufficient to restore proteomic homeostasis, keeping pathways related to inflammation, immune responses, and structural reorganization activated.

Additional proteins of relevance include molecules involved in energy metabolism (such as creatine kinase isoforms), extracellular matrix proteins, cellular stress modulators, and calcium-binding proteins, which together reflect the physiopathological adaptations typical of chronic cardiomyopathy associated with resistant strains.

In summary, the chronic phase of VL-10 infection is characterized by persistent immune activation, ongoing structural and metabolic damage, continuous tissue remodeling, and limited therapeutic modulation. These findings reinforce the impact of BNZ resistance and parasite genetic variability on disease progression and on the systemic proteomic profile.

### 3.5. Differentially Abundant Proteins in Be-78 vs. VL-10 Strains

**Figure 6:**
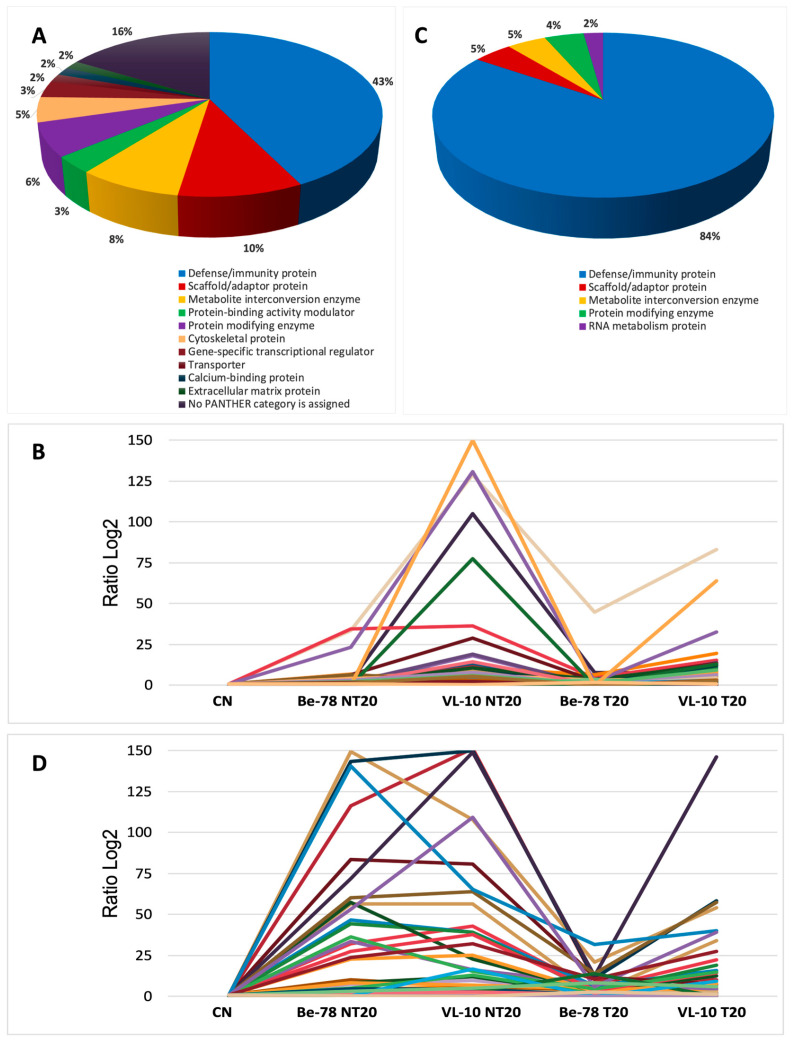
Comparative analysis of differentially abundant serum proteins between the Be-78 and VL-10 strains during the acute and chronic phases of *Trypanosoma cruzi* infection in a mouse model. (**A**) Functional classification of proteins modulated during the acute phase. (**B**) Protein abundance variation in the acute phase expressed as log_2_ fold change (Be-78 vs. VL-10). (**C**) Functional classification of proteins modulated during the chronic phase. (**D**) Protein abundance variation in the chronic phase expressed as log_2_ fold change (Be-78 vs. VL-10).

In the acute phase of infection, 51 proteins were identified as shared between the Be-78 and VL-10 strains (Figure S3 and Figure 6; Table S9), corresponding to approximately 10% of the total differentially abundant proteins. This set consisted primarily of complement system components, heavy-, kappa-, and lambda-chain immunoglobulins, and inflammatory proteins, forming a systemic inflammatory core common to both infections.

Among the shared proteins, C1qA, C1qB, and C6 stood out, demonstrating robust activation of the classical and terminal complement pathways in both strains, although with greater magnitude in VL-10, as illustrated by the markedly higher C6 levels in this strain. Shared inflammatory and immunomodulatory proteins—including BPI fold-containing protein (BPI), Cathepsin S, CD5 antigen-like, and Histocompatibility 2 Q region locus 4—suggest concurrent activation of pathways related to recognition, processing, and antigen presentation.

Metabolic and systemic response markers such as Hemopexin, Phospholipid Transfer Protein (PLTP), and Trimethylguanosine Synthase were also consistently detected in both strains, indicating that adjustments in lipid metabolism and heme/iron transport form part of the shared inflammatory axis. However, not all shared proteins exhibited the same direction or intensity of modulation: for instance, Apolipoprotein C-III showed slight reductions across several groups rather than increases, whereas proteins such as Factor XIII A chain and Fibronectin exhibited distinct behavior between strains and conditions.

The cellular injury marker L-lactate dehydrogenase A chain (LDH-A) showed an asymmetric pattern between strains. In Be-78, LDH-A decreased in NT20 and dropped further after treatment, whereas in VL-10 it exhibited a pronounced increase in both NT20 and T20, indicating more severe tissue damage and metabolic dysfunction in this strain. Thus, although LDH-A was shared between the infections, its modulation was far more pronounced in VL-10, reinforcing its more aggressive phenotype.

Overall, most of the shared proteins were upregulated in the NT20 group in at least one of the strains, with therapeutic effects being more evident in Be-78, where BNZ (T20) consistently reduced the abundance of multiple complement components, immunoglobulins, and inflammatory markers. In VL-10, although some reduction occurred following treatment, many markers remained substantially elevated compared with controls, reflecting the lower therapeutic responsiveness of this strain.

In the chronic phase, 70 proteins were shared between Be-78 and VL-10 across all experimental conditions (CN, NT20, and T20) (Figure 6; Table S10). This set indicates that, even at later stages, both infections maintain a common axis of persistent immune response, tissue remodeling, and metabolic alterations associated with disease chronicity.

Among shared immunological components, several members of the complement system stood out, including C1qA, C1qB, C1qC, and C1s-1, as well as C-reactive protein (CRP), a classical marker of chronic inflammation. A broad repertoire of heavy-, kappa-, and lambda-chain immunoglobulins remained modulated in both strains, indicating continued humoral activation during chronicity; however, the magnitude of modulation was generally higher in VL-10, particularly for kappa chains and the J chain.

Structural remodeling and tissue-support proteins were also widely represented among shared components, including Dystrophin, Myosin XVIIIb, Alpha-adducin, Centromere-associated protein E, EGF-containing fibulin-like protein 1, and cytoskeletal Keratin 42. The presence and modulation of these proteins suggest prolonged alterations in the cytoskeleton, extracellular matrix, and tissue organization, with more pronounced effects in animals infected with the VL-10 strain, where abundance levels frequently exceeded those observed in groups infected with Be-78.

Metabolic and cellular injury markers reinforced this asymmetry between strains. LDH-A showed dramatic increases in VL-10 NT20 group and remained elevated after treatment, whereas in Be-78 groups its abundance was reduced or close to control levels. Similarly, Pyrin, a protein associated with inflammasome activation, was elevated in both strains but reached much higher levels in VL-10-infected groups, suggesting more intense chronic inflammatory activation in this model.

**Figure 7:**
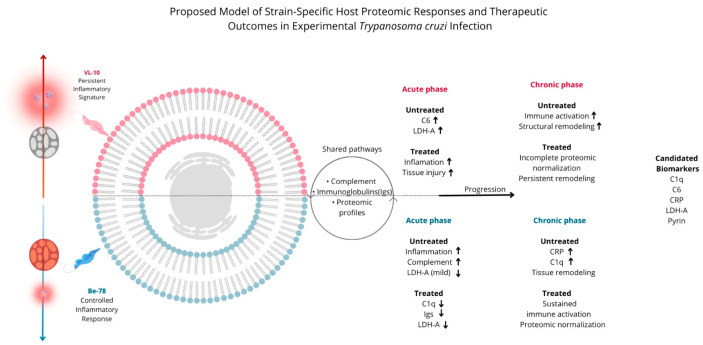
Integrative biological model linking *Trypanosoma cruzi* strain variability to host proteomic signatures and therapeutic response. Genetic diversity among parasite strains drives distinct systemic proteomic adaptations. The benznidazole-susceptible Be-78 strain promotes a controlled inflammatory response with proteomic normalization after treatment, whereas the resistant VL-10 strain is characterized by sustained inflammation and diminished therapeutic responsiveness. Shared pathways include complement activation, inflammatory signaling, immunoglobulin modulation, energy metabolism, and tissue remodeling. Representative proteins—including C1q, C6, C-reactive protein, LDH-A, and Pyrin—highlight potential serum biomarkers for monitoring disease progression and treatment outcomes.

Taken together, chronic-phase data show that although there is a shared set of proteins in the serum of mice infected with between Be-78 and VL-10 strains, the magnitude of proteomic modulation is substantially greater in animals infected with the VL-10 strain, with high abundance of inflammatory, structural, and metabolic proteins. After BNZ treatment (T20), Be-78 infected mice exhibited a more pronounced reduction in most shared proteins, approaching the profile of the control group, whereas VL-10 infected groups maintained persistently high levels, indicating ongoing systemic inflammation, active tissue remodeling, and limited therapeutic response (Figure 7).

## 4. Discussion

### 4.1. Modulation of the Most Abundant Proteins and Biological Implications

Comparative evaluation of differentially abundant serum proteins between the Be-78 (BNZ-susceptible) and VL-10 (BNZ-resistant) strains revealed patterns strongly associated with the dynamics of *T. cruzi* infection and with intrinsic differences in virulence and therapeutic responsiveness [1,2,9,25]. In the acute phase, both strains showed a pronounced increase in inflammatory components, immunoglobulins, and complement system proteins; however, the magnitude of these changes differed markedly, reflecting strain-specific sensitivity to BNZ and intrinsic capacity to induce systemic inflammation.

Key innate immunity proteins such as Complement C1qA/B and Complement C6 displayed extremely elevated levels in both NT20 groups, with a striking predominance in VL-10, whose values far exceeded those observed in Be-78. This pattern aligns with classical and alternative complement hyperactivation during the acute phase [19], and with studies reporting exacerbated responses in more virulent strains [8,26]. The strong increase in C6, observed at much higher magnitudes in VL-10, reinforces the involvement of the terminal complement pathway, potentially contributing to cell lysis and tissue injury—phenomena previously demonstrated in cardiac models of Chagas cardiomyopathy [23,27].

The robust elevation of immunoglobulins—including multiple heavy-, kappa-, and lambda-chain variants—reflects the polyclonal expansion characteristic of *T. cruzi* infection, which is more intense in VL-10. The presence of CD5 antigen-like, BPI, and Cathepsin S indicates simultaneous activation of immune recognition, antigen processing, and modulation of the adaptive response, mechanisms amplified during early infection [2,19,28].

Metabolic and systemic response markers also exhibited critical differences between strains. The pronounced elevation of LDH-A in VL-10 suggests greater systemic cellular damage, consistent with myocardial and skeletal muscle injury described in aggressive experimental models [23,29,30]. In contrast, Be-78 showed moderate increases in NT20 and clear reduction after treatment, reflecting effective restoration of the proteome following BNZ exposure—consistent with its higher drug sensitivity [21,24].

Lipid-transport proteins such as phospholipid transfer protein (PLTP) and structural proteins such as fibronectin also showed distinct patterns, reflecting extracellular matrix alterations and compensatory responses to tissue injury [23,31]. Taken together, these findings reinforce that the most abundant proteins capture, in an integrated manner, systemic inflammation, tissue injury, structural remodeling, and therapeutic efficacy—in agreement with previous studies [2,18,19].

The elevation of hemopexin observed in this study can be interpreted within the framework of its established physiological role in extracellular heme scavenging and protection against heme-mediated oxidative stress. Hemopexin is a high-affinity heme-binding acute-phase protein that neutralizes the pro-oxidant and pro-inflammatory effects of free heme released during tissue injury and systemic inflammation, functioning as a key compensatory host defense mechanism [16]. In infectious conditions characterized by hemolysis, such as malaria, dysregulation of the free heme–hemopexin axis has been associated with tissue injury and inflammatory activation, highlighting the biological relevance of this pathway [32]. In the context of *T. cruzi* infection, the increased abundance of hemopexin is therefore consistent with activation of heme-related protective pathways and systemic inflammatory responses, broadening the mechanistic interpretation of our proteomic findings.

### 4.2. Proteins Exclusively Identified in Each Condition

The analysis of proteins uniquely identified in each group revealed critical elements underlying the differential pathophysiology between the strains. In the acute phase, both Be-78 and VL-10 exhibited exclusive proteins associated with immune responses, but their specific profiles revealed distinct signatures.

VL-10 displayed, either exclusively or markedly upregulated, proteins such as C6, highly variable immunoglobulins, and molecules associated with cell adhesion and cellular injury, reflecting reduced susceptibility to treatment and accentuated tissue damage. This pattern is consistent with studies documenting early structural disorganization, sarcomeric disruption, and exacerbated inflammation in more aggressive strains [23,26].

In the chronic phase, VL-10 exhibited the highest number of exclusive proteins, including structural components such as dystrophin, myosin XVIIIb, alpha-adducin, ECM-related proteins such as fibulin-like proteins, and inflammatory markers such as Pyrin. These findings reinforce that the resistant strain sustains progressive chronic tissue remodeling—an observation coherent with studies of cardiac remodeling during chronic *T. cruzi* infection [33].

Be-78, in contrast, retained only a few exclusive targets following BNZ treatment, indicating substantial proteomic normalization consistent with its high therapeutic sensitivity [24]. This pattern suggests that BNZ efficacy reduces not only parasite burden but also immune activation and systemic damage—a phenomenon partially observed in both clinical and experimental studies [21].

The presence of exclusive metabolic, cytoskeletal, and inflammatory proteins in chronic VL-10 infection (including Nebulin, Myosin, LDH-A, centromeric components, and ECM-related proteins) reinforces the persistent nature of tissue injury and the strain’s resistance to restorative mechanisms. Such a signature is rarely observed in serum-based studies, being more commonly reported in direct analyses of cardiac tissue [23,34], underscoring the severity of the pathophysiology induced by the resistant strain.

### 4.3. Observed Proteomic Profiles

The proteomic profiles obtained revealed robust and clearly distinct signatures between the strains. Be-78 triggers an intense inflammatory process in the acute phase, but with a markedly greater capacity for reversal following BNZ treatment, whereas VL-10 sustains a more severe inflammatory and destructive state even after therapy. Previous studies have demonstrated that variability among DTUs influences immune evasion capacity, BNZ resistance, and the aggressiveness of cardiac damage [7,26], and our results confirm these findings at a systemic proteomic level.

VL-10 exhibited extreme increases in complement components (C1qA/B/C, C6), highly variable immunoglobulins, markers of muscle injury (LDH-A, dystrophin, nebulin), and structural proteins associated with the cytoskeleton and extracellular matrix. This set of proteins characterizes a hyper-inflammatory, tissue-damaging, and metabolically dysfunctional phenotype, consistent with aggressive chronic cardiomyopathy [23,29,30].

The marked increase in Pyrin in VL-10 is particularly noteworthy. Pyrin is a key regulator of the inflammasome, and its activation is associated with IL-1β release, necroinflammation, and progression of Chagas cardiomyopathy [35]. The magnitude of its modulation suggests that VL-10 activates the Pyrin/NLRP3–IL-1β axis more strongly, contributing to persistent inflammation and ongoing tissue remodeling.

Be-78, in contrast, although it exhibits a strong initial humoral and inflammatory response, demonstrates coordinated proteomic reversal following BNZ treatment, including normalization of LDH-A, C1q, C6, and several immunoglobulins—consistent with an effective therapeutic response [21,24].

These findings reinforce that VL-10 induces a more aggressive, sustained, and destructive systemic response, whereas Be-78 exhibits a more controlled and reversible inflammatory process—providing a clear mechanistic basis for the distinct therapeutic outcomes observed (Figure 7).

### 4.4. Potential Biomarkers of Tissue Injury and Therapeutic Response

The lack of reliable serum biomarkers remains one of the major challenges in Chagas disease [1,3]. Our findings advance this field by identifying multi-biomarker panels associated with tissue injury, inflammation, complement activation, and therapeutic response.

Biomarkers of muscle and cardiac injury.

The marked elevation of LDH-A in animals infected with the VL-10 strain, together with the serum presence of dystrophin and nebulin, reinforces the occurrence of true muscle fiber rupture, rather than simply metabolic stress, as documented in cardiac studies [23]. This combination could represent a robust panel for identifying highly aggressive infections.

Inflammatory and immunological biomarkers.

The overexpression of C1qA/B/C, C6, and CRP confirms persistent complement activation and systemic inflammation, phenomena described in patients with Chagas cardiomyopathy [23,29]. The polyclonal expansion of κ and λ immunoglobulins follows the pattern observed in both patients and experimental models [36,37], but their differential profiles between strains provide additional diagnostic value.

Inflammasome-related biomarkers.

The increased abundance of Pyrin, especially in VL-10 infections, suggests sustained activation of Pyrin/NLRP3 pathways and supports its potential as a marker of sterile inflammation and cardiac dysfunction [35,38].

Biomarkers of benznidazole response.

Coordinated normalization of LDH-A, C1q, immunoglobulins, and structural proteins was observed only in Be-78 infections, indicating that this set may be used to assess effective BNZ response, consistent with both clinical and experimental observations [24].

Thus, an integrated biomarker panel composed of: LDH-A, dystrophin, nebulin (muscle injury/cardiomyopathy); C1q, C6, CRP (inflammation and complement activation); variable κ/λ Ig chains (humoral activation); and Pyrin (inflammasome activation) could represent a powerful approach for characterizing disease progression, aggressiveness, and therapeutic response in Chagas disease (Figure 7).

The serum proteomic profiles identified allow functional association of specific protein sets with potential utility as biomarkers of infection phase and strain variability in *T. cruzi*. Markers such as C1q, C6, and immunoglobulins were more strongly linked to the acute phase, reflecting inflammatory and complement activation. In contrast, proteins associated with persistent muscle injury and tissue remodeling, including nebulin, dystrophin, and LDH-A, were more closely related to the chronic phase. Sustained elevation of LDH-A, C6, and Pyrin, particularly in VL-10 infection, further suggests their potential as indicators of greater biological aggressiveness and strain-specific differences. Although not definitive, these associations support the role of these proteomic signatures as candidate serum biomarkers for monitoring infection dynamics.

In this study, we used total non-depleted serum, a well-known approach as referenced in [13] that provides a broader view of the proteomic profile. While this choice allows for the identification of many relevant proteins, we acknowledge that it may limit the detection of cytokines or lower molecular weight proteins. This is a limitation of the technique and does not mean these molecules are absent from the sample.

## 5. Conclusions

This serum proteomic analysis demonstrates that *Trypanosoma cruzi* genetic variability is a major determinant of systemic inflammation, tissue injury, and responsiveness to benznidazole therapy. Infection with the benznidazole-sensitive Be-78 strain was associated with broad proteomic normalization following treatment, whereas mice infected with the benznidazole-resistant VL-10 strain exhibited sustained elevation of inflammatory mediators, complement components, immunoglobulins, and structural proteins during both acute and chronic phases, consistent with persistent inflammation and reduced therapeutic responsiveness.

Several key molecules, including C1q subunits, C6, C-reactive protein, LDH-A, Pyrin, and structural proteins such as dystrophin, nebulin, adducin, and myosins, emerge as promising serum biomarkers of tissue damage, ongoing inflammation, and therapeutic response. Notably, the incomplete normalization of these markers in VL-10-infected mice suggests a distinct and measurable molecular signature associated with treatment resistance.

Collectively, these findings highlight the power of serum proteomics to uncover biological signatures linked to parasite virulence, inflammatory burden, and drug efficacy. Our results support the development of multi-marker serum panels for monitoring Chagas disease progression and treatment outcomes and underscore the importance of incorporating *T. cruzi* genetic diversity into the design of diagnostic tools and therapeutic strategies.

## Figures and Tables

**Table 1:** UHPLC-MS/MS Indicators of the Serum Proteome in the Acute Phase.

Indicator	Be-78	VL-10
MS	207.013	205.877
MS/MS	189.214	191.622
Peptide-Spectrum Matches (PSM)	62.311	63.736
Peptide sequences	3.736	3.796
Protein groups	384	379
Protein	517	506
Proteins (unique peptides)	251 (>2); 82 (=2); 184 (=1)	239 (>2); 80 (=2); 187 (=1)

**Table 2:** UHPLC-MS/MS Indicators of the Serum Proteome in the Chronic.

Indicator	Be-78	VL-10
MS	245,585	242,042
MS/MS	172,031	178,275
Peptide-Spectrum Matches (PSM)	39,724	44,640
Peptide sequences	2322	2554
Protein groups	267	308
Protein	380	422
Proteins (unique peptides)	188 (>2); 61 (=2); 131 (=1)	200 (>2); 75 (=2); 147 (=1)

## Data Availability

The original contributions presented in this study are included in the article/Supplementary Material. Further inquiries can be directed to the corresponding author.

## Supplementary Materials

The following supporting information can be downloaded at: https://www.mdpi.com/article/10.3390/microorganisms14030588/s1, Table S1: Complete list of proteins identified in the acute phase; Table S2: Complete list of proteins identified in the chronic phase; Table S3: Description of the top 10 most abundant proteins present in the CN, NT20, and T20 subgroups during experimental infection in the acute and chronic phases with the different *T. cruzi* strains Be-78 and VL-10; Table S4: Description of proteins found exclusively in the CN, NT20, and T20 subgroups of mice experimentally infected with the Be-78 and VL-10 *T. cruzi* strains during the acute and chronic phases; Table S5: Differentially abundant proteins in the Be-78 strain during the acute phase; Table S6; Differentially abundant proteins in the Be-78 strain during the chronic phase; Table S7: Differentially abundant proteins in the VL-10 strain during the acute phase; Table S8: Differentially abundant proteins in the VL-10 strain during the chronic phase; Table S9: Proteins shared between the Be-78 and VL-10 strains in the acute phase; Table S10: Proteins shared between the Be-78 and VL-10 strains in the chronic phase; Figure S1: Analysis of differentially abundant serum proteins in the Be-78 strain during the acute and chronic phases of *T. cruzi* infection. (A) Heatmap of differentially abundant proteins in the acute phase. (B) Heatmap of differentially abundant proteins in the chronic phase; Figure S2: Analysis of differentially abundant serum proteins in the VL-10 strain during the acute and chronic phases of *T. cruzi* infection. (A) Heatmap of differentially abundant proteins in the acute phase. (B) Heatmap of differentially abundant proteins in the chronic phase; Figure S3: Comparative analysis of differentially abundant serum proteins between the Be-78 and VL-10 strains during the acute and chronic phases of *Trypanosoma cruzi* infection in a mice model. (A) Heatmap depicting differentially abundant serum proteins in the acute phase (Be-78 vs. VL-10). (B) Heatmap depicting differentially abundant serum proteins in the chronic phase (Be-78 vs. VL-10).

## Abbreviations

The following abbreviations are used in this manuscript:

BNZ	Benznidazol
DTU	Discrete Typing Unit
LDH-A	L-lactate dehydrogenase A chain
GO	Gene Ontology
qPCR	Quantitative Polymerase Chain Reaction
MS	Mass spectrometry
MS/MS	Tandem mass spectrometry
