# Supplementary material for: Serum Proteomics Provides Novel Biomarkers of Inflammation, Tissue Injury, and Therapeutic Response in Experimental Chagas Disease
Source: Microorganisms. 2026 Mar 5;14(3):588. doi: 10.3390/microorganisms14030588 (PMC13028842; doi:10.3390/microorganisms14030588)
Supplement: Supplementary file 1 [file microorganisms-14-00588-s001.zip › microorganisms-4103234-supplementary-1.pdf]

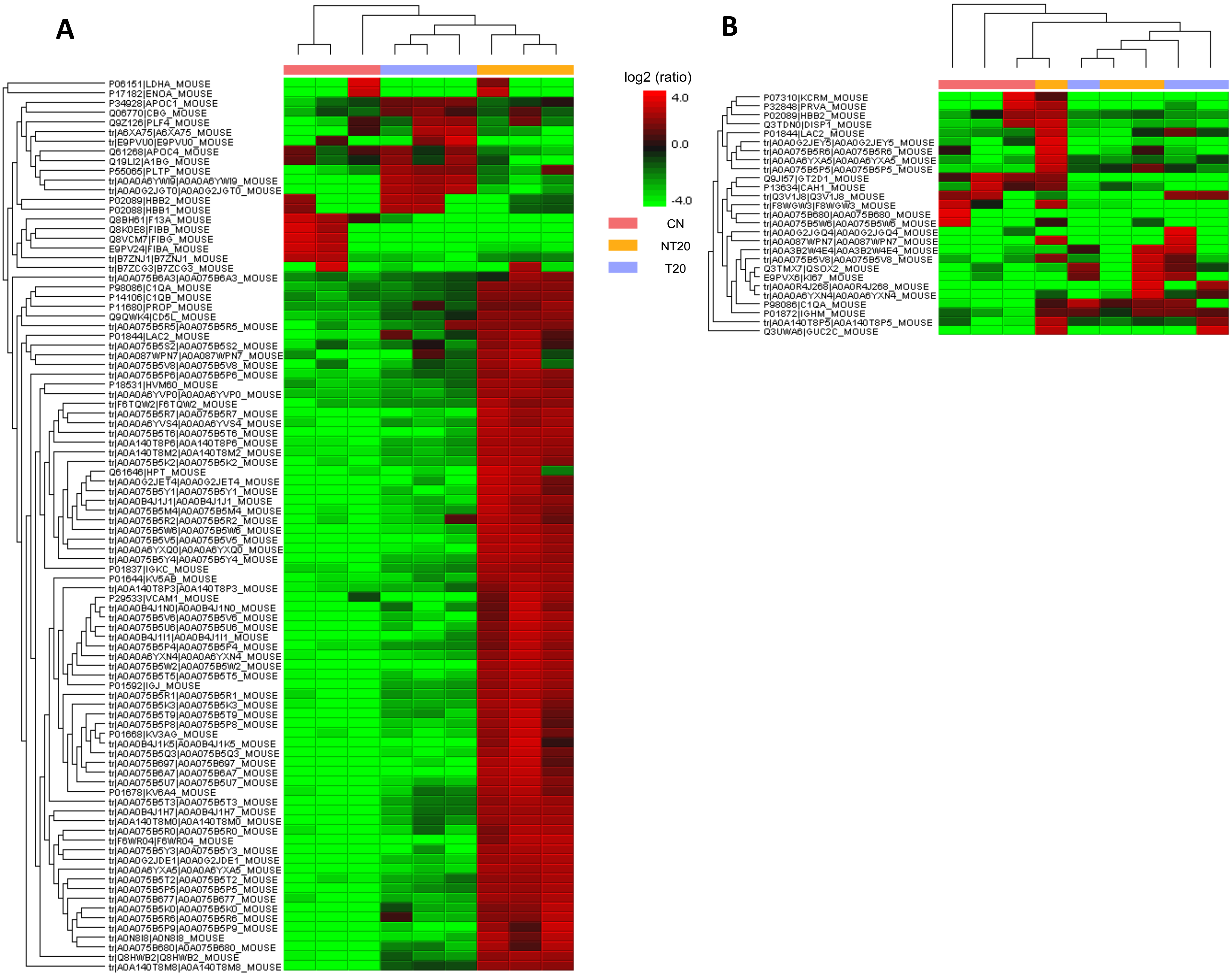

Figure S1. Analysis of differentially abundant serum proteins in the Be-78 strain during the acute and chronic phases of *T. cruzi* infection. (A) Heatmap of differentially abundant proteins in the acute phase. (B) Heatmap of differentially abundant proteins in the chronic phase.

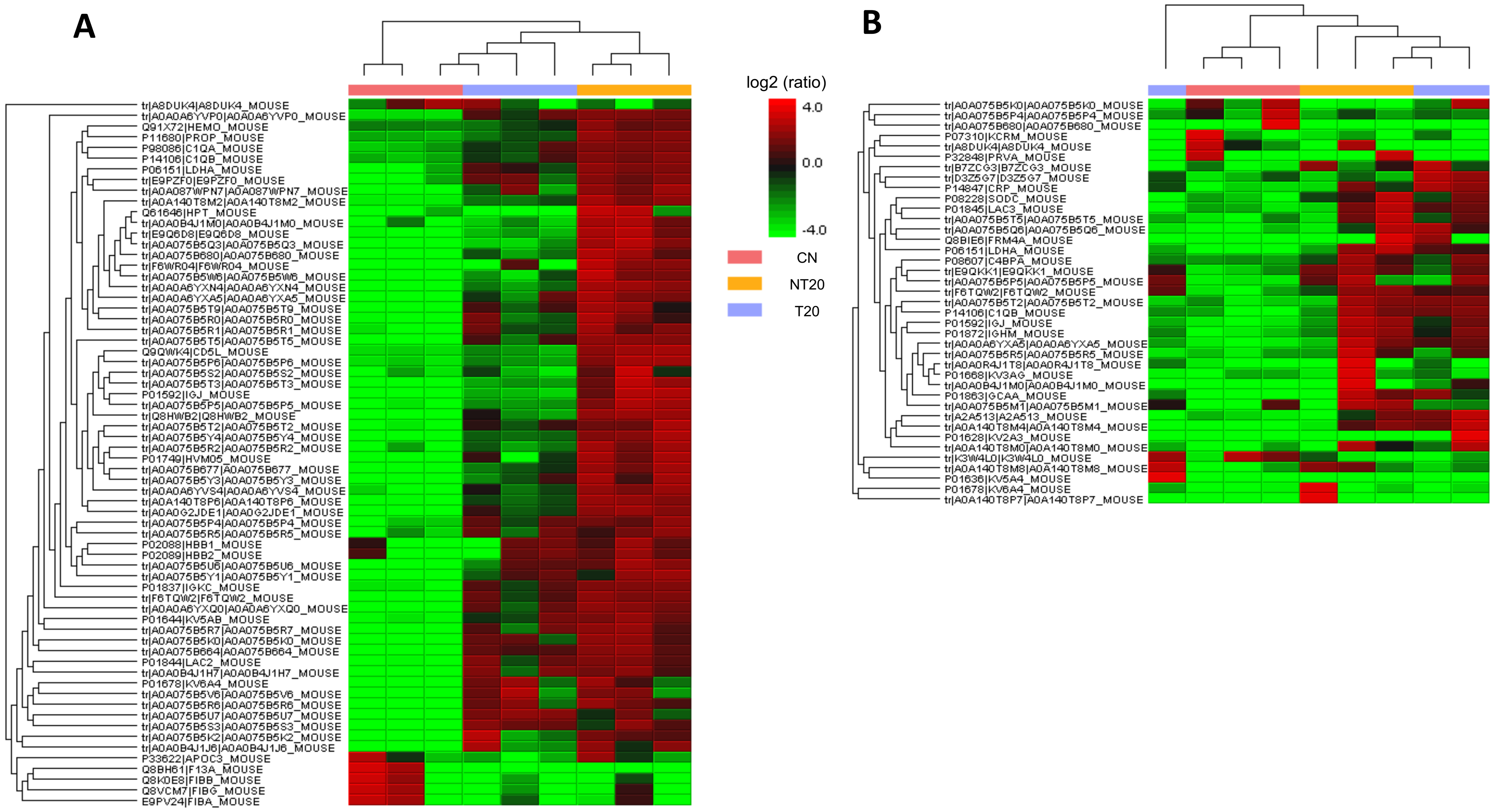

Figure S2. Analysis of differentially abundant serum proteins in the VL-10 strain during the acute and chronic phases of *T. cruzi* infection. (A) Heatmap of differentially abundant proteins in the acute phase. (B) Heatmap of differentially abundant proteins in the chronic phase.

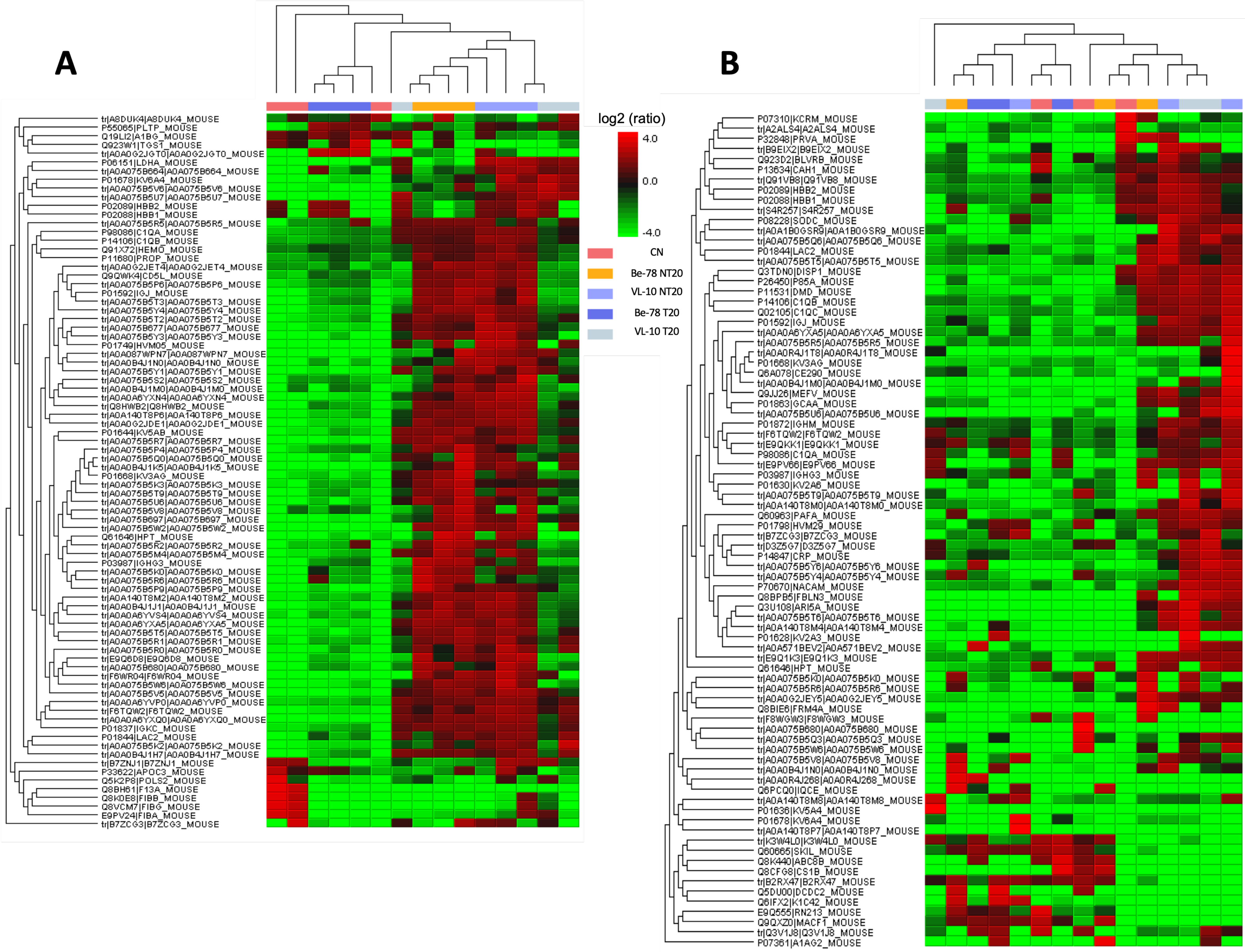

Figure S3. Comparative analysis of differentially abundant serum proteins between the Be-78 and VL-10 strains during the acute and chronic phases of *Trypanosoma cruzi* infection in a mice model. (A) Heatmap depicting differentially abundant serum proteins in the acute phase (Be-78 vs. VL-10). (B) Heatmap depicting differentially abundant serum proteins in the chronic phase (Be-78 vs. VL-10).
