# Supplementary material for: Serum Proteomics Provides Novel Biomarkers of Inflammation, Tissue Injury, and Therapeutic Response in Experimental Chagas Disease
Source: Microorganisms. 2026 Mar 5;14(3):588. doi: 10.3390/microorganisms14030588 (PMC13028842; doi:10.3390/microorganisms14030588)
Supplement: Supplementary file 1 [file microorganisms-14-00588-s001.zip › Tables. docx.pdf]

Table S1. Complete list of proteins identified in the acute phase

N°	Access	Description	Coverage (%)	Identified Peptides	Unique Peptides	Molecular Mass (Da)
1	P68033	Actin, alpha cardiac muscle 1	30	10	1	42019
2	P68134	Actin, alpha skeletal muscle	30	10	1	42051
3	P60710	Actin, cytoplasmic 1	41	13	2	41737
4	P63260	Actin, cytoplasmic 2	41	13	2	41793
5	Q8VHN7	Adhesion G-protein coupled receptor V1	0	2	2	687469
6	B8JJE0	Adhesion G-protein-coupled receptor V1	0	2	2	687618
7	Q60994	Adiponectin	15	3	3	26809
8	O89020	Afamin	60	38	37	69379
9	P07724	Albumin	90	73	73	68693
10	Q91VB8	Alpha globin 1	92	9	9	15112
11	A0A1W2P788	alpha-1,2-Mannosidase (Fragment)	3	2	2	82043
12	Q60590	Alpha-1-acid glycoprotein 1	35	8	7	23895
13	P07361	Alpha-1-acid glycoprotein 2	13	2	1	23843
14	P22599	Alpha-1-antitrypsin 1-2	62	25	10	45975
15	Q00897	Alpha-1-antitrypsin 1-4	51	23	5	45998
16	Q00898	Alpha-1-antitrypsin 1-5	24	12	1	45891
17	Q19LI2	Alpha-1B-glycoprotein	58	26	26	56554
18	Q61247	Alpha-2-antiplasmin	54	22	22	54972
19	P29699	Alpha-2-HS-glycoprotein	46	12	9	37326
20	P00687	Alpha-amylase 1	26	11	11	57644
21	P17182	Alpha-enolase	13	5	3	47141
22	Q3UTR7	Angiotensinogen	28	10	10	52670
23	A2RT91	Ankyrin and armadillo repeat-containing protein	1	1	1	165289

24	P32261	Antithrombin-III	70	34	34	52004
25	Q00623	Apolipoprotein A-I	80	35	34	30616
26	P09813	Apolipoprotein A-II	48	5	5	11309
27	P06728	Apolipoprotein A-IV	83	35	34	45029
28	E9Q414	Apolipoprotein B-100	32	117	117	509437
29	P34928	Apolipoprotein C-I	39	4	4	9696
30	Q05020	Apolipoprotein C-II	33	3	3	10741
31	A0A0R4J1N3	Apolipoprotein C-III	59	6	1	10922
32	E9QP56	Apolipoprotein C-III	42	6	1	15163
33	P33622	Apolipoprotein C-III	59	7	2	10982
34	Q61268	Apolipoprotein C-IV	26	4	4	14288
35	P51910	Apolipoprotein D	40	7	7	21530
36	P08226	Apolipoprotein E	66	23	22	35867
37	Q9Z1R3	Apolipoprotein M	36	8	8	21273
38	G3X9D6	Apolipoprotein N	17	3	3	27965
39	Q01339	Beta-2-glycoprotein 1	62	24	24	38619
40	P01887	Beta-2-microglobulin	71	6	6	13779
41	P21550	Beta-enolase	7	3	1	47025
42	A8DUK4	Beta-globin	95	17	6	15748
43	A0A0R4J131	Biotinidase	4	2	2	59279
44	Q8CIF4	Biotinidase	4	2	2	58154
45	P15327	Bisphosphoglycerate mutase	7	1	1	29978
46	B7ZCG3	BPI fold-containing family A member 2 (Fragment)	41	6	6	22321
47	P07743	BPI fold-containing family A member 2	37	6	6	24753
48	P08607	C4b-binding protein	29	10	10	51524
49	P13634	Carbonic anhydrase 1	30	5	5	28331
50	P00920	Carbonic anhydrase 2	62	12	12	29033
51	P16015	Carbonic anhydrase 3	38	7	7	29366

52	P23953	Carboxylesterase 1C	65	29	20	61056
53	D3Z5G7	Carboxylic ester hydrolase	42	21	12	62197
54	Q9JHH6	Carboxypeptidase B2	29	10	10	48871
55	Q9JJN5	Carboxypeptidase N catalytic chain	32	11	11	51845
56	Q9DBB9	Carboxypeptidase N subunit 2	33	17	17	60479
57	Q9WVJ3	Carboxypeptidase Q	10	3	3	51813
58	P10605	Cathepsin B	5	1	1	37280
59	F6WR04	Cathepsin S	17	4	4	38631
60	O70370	Cathepsin S	17	4	4	38475
61	Q9Z121	C-C motif chemokine 8	29	2	2	11017
62	Q9QWK4	CD5 antigen-like	55	18	18	38863
63	E9Q3P4	Centromere protein F	0	2	1	342472
64	Q6ZQ06	Centrosomal protein of 162 kDa	1	1	1	160854
65	E9Q5A8	Centrosome-associated protein CEP250	1	1	1	278838
66	A3KGJ7	Centrosome-associated protein CEP250	1	1	1	278968
67	Q60952	Centrosome-associated protein CEP250	1	1	1	276811
68	E9PZD8	Ceruloplasmin	63	59	59	124211
69	Q61147	Ceruloplasmin	65	59	59	121151
70	Q9EQI5	Chemokine subfamily B Cys-X-Cys	19	2	2	12252
71	Q03311	Cholinesterase	4	2	2	68462
72	P05208	Chymotrypsin-like elastase family member 2A	8	1	1	28914
73	E9Q2G2	Clusterin (Fragment)	18	8	1	23651
74	Q06890	Clusterin	36	18	11	51656
75	O88783	Coagulation factor V	2	4	4	247228
76	O88947	Coagulation factor X	33	13	13	54018
77	Q3U3V1	Coagulation factor X	32	13	13	55255
78	Q80YC5	Coagulation factor XII	20	9	9	65701
79	Q8BH61	Coagulation factor XIII A chain	7	4	4	83207

80	Q07968	Coagulation factor XIII B chain	30	14	14	76195
81	P11087	Collagen alpha-1(I) chain	5	5	5	138033
82	J3QQ16	Collagen, type VI, alpha 3	1	1	1	288693
83	A0A087WS16	Collagen, type VI, alpha 3	1	1	1	288691
84	E9PWQ3	Collagen, type VI, alpha 3	0	1	1	353932
85	P98086	Complement C1q subcomponent subunit A	29	6	6	25974
86	P14106	Complement C1q subcomponent subunit B	33	6	6	26717
87	Q02105	Complement C1q subcomponent subunit C	20	4	4	25992
88	Q8CG16	Complement C1r-A subcomponent	22	12	11	80073
89	E9Q6C2	Complement C1s-1 subcomponent	22	11	11	77500
90	Q8CG14	Complement C1s-1 subcomponent	22	11	11	76858
91	B8JJM9	Complement C2	8	4	1	54270
92	B8JJN2	Complement C2	6	4	1	69685
93	P21180	Complement C2	5	4	1	84742
94	P01027	Complement C3	82	125	123	186483
95	P01029	Complement C4-B	53	72	72	192914
96	P06684	Complement C5	17	21	21	188877
97	Q91X70	Complement component 6	9	4	4	86631
98	E9Q6D8	Complement component C6	7	4	4	104624
99	Q8K182	Complement component C8 alpha chain	41	18	18	66080
100	Q8BH35	Complement component C8 beta chain	27	12	12	66229
101	Q9DAC2	Complement component C8 gamma chain	61	8	8	18946
102	Q8VCG4	Complement component C8 gamma chain	51	8	8	22508
103	A0A0R4J032	Complement component C9	27	15	15	63426
104	P06683	Complement component C9	27	15	15	62002
105	B8JJN0	Complement factor B	33	44	9	142325
106	P03953	Complement factor D	40	6	6	28057
107	E9Q8I0	Complement factor H	65	65	51	141271

108	P06909	Complement factor H	66	65	51	139137
109	Q61406	Complement factor H-related 1	11	4	3	38443
110	Q4LDF6	Complement factor H-related 2	39	10	2	37932
111	E9Q8B6	Complement factor H-related 4	15	13	6	92839
112	E9Q8B5	Complement factor H-related 4	14	13	6	99438
113	Q61129	Complement factor I	49	25	25	67261
114	Q06770	Corticosteroid-binding globulin	53	19	19	44769
115	P14847	C-reactive protein	22	4	4	25360
116	P07310	Creatine kinase M-type	36	12	12	43045
117	Q8CFZ6	C-type lectin domain family 3, member b	20	3	3	22255
118	Q9CPY7	Cytosol aminopeptidase	2	1	1	56141
119	Q8BL66	Early endosome antigen 1	1	2	2	160914
120	Q8BPB5	EGF-containing fibulin-like extracellular matrix protein 1	5	2	2	54953
121	P10126	Elongation factor 1-alpha 1	6	3	3	50114
122	Q01279	Epidermal growth factor receptor	16	16	16	134853
123	A0A2I3BRQ1	Expressed sequence AI182371	15	5	5	40054
124	F8WI14	Extracellular matrix protein 1	10	5	5	62718
125	Q61508	Extracellular matrix protein 1	10	5	5	62832
126	O09164	Extracellular superoxide dismutase [Cu-Zn]	6	1	1	27392
127	F2Z4A3	FAT atypical cadherin 1	0	1	1	506271
128	A0A1L1SQU7	FAT atypical cadherin 1	0	1	1	512199
129	A0A087WRT4	FAT atypical cadherin 1	0	1	1	507536
130	P04117	Fatty acid-binding protein, adipocyte	16	2	2	14650
131	P12710	Fatty acid-binding protein, liver	9	1	1	14246
132	Q9QXC1	Fetuin-B	54	15	15	42713
133	E9PV24	Fibrinogen alpha chain	54	36	35	87429
134	Q8K0E8	Fibrinogen beta chain	77	39	39	54753
135	Q8VCM7	Fibrinogen gamma chain	78	33	33	49391

136	Q3UER8	Fibrinogen gamma chain	77	33	33	50349
137	B9EHT6	Fibronectin	54	83	83	250295
138	A0A087WSN6	Fibronectin	53	83	83	253009
139	Q08879	Fibulin-1	12	6	6	78033
140	O70165	Ficolin-1	5	2	2	36298
141	E9PZC3	Flavin reductase (NADPH)	23	4	4	26647
142	Q923D2	Flavin reductase (NADPH)	28	4	4	22197
143	P05064	Fructose-bisphosphate aldolase A	26	7	7	39356
144	A6ZI44	Fructose-bisphosphate aldolase	23	7	7	45120
145	P13020	Gelsolin	57	36	34	85942
146	P46412	Glutathione peroxidase 3	33	8	7	25424
147	S4R257	Glyceraldehyde-3-phosphate dehydrogenase (Fragment)	29	6	1	29939
148	P01898	H-2 class I histocompatibility antigen, Q10 alpha chain	56	16	8	37251
149	Q61646	Haptoglobin	47	17	17	38752
150	Q504P4	Heat shock cognate 71 kDa protein	2	1	1	68779
151	P63017	Heat shock cognate 71 kDa protein	2	1	1	70871
152	P02088	Hemoglobin subunit beta-1	95	17	2	15840
153	P02089	Hemoglobin subunit beta-2	95	17	7	15878
154	P04443	Hemoglobin subunit beta-H0	7	1	1	16384
155	P04444	Hemoglobin subunit beta-H1	7	1	1	16494
156	Q91X72	Hemopexin	70	33	32	51318
157	P49182	Heparin cofactor 2	49	19	19	54497
158	Q9R098	Hepatocyte growth factor activator	14	7	7	70568
159	E0CXN0	Hepatocyte growth factor-like protein	6	3	2	79715
160	P26928	Hepatocyte growth factor-like protein	6	3	2	80619
161	A0A0R4J039	Histidine-rich glycoprotein	34	19	19	60439
162	Q9ESB3	Histidine-rich glycoprotein	34	19	19	59163
163	O19441	Histocompatibility 2, Q region locus 1	17	6	2	41322

164	E9PX63	Histocompatibility 2, Q region locus 1	18	6	2	38616
165	Q8HWB2	Histocompatibility 2, Q region locus 4	38	12	4	39618
166	Q6S9I3	HMW kininogen-II	22	10	6	71272
167	Q3V1J8	Hyaluronan-binding protein 2	11	4	4	57326
168	E9Q092	Hyaluronan-binding protein 2	11	4	4	61298
169	E9QM92	Hyaluronan-binding protein 2	11	4	4	61823
170	Q8K0D2	Hyaluronan-binding protein 2	11	4	4	62357
171	A0A075B5P4	Ig gamma-1 chain C region secreted form (Fragment)	58	12	4	35752
172	A0A0A6YWR2	Ig gamma-1 chain C region secreted form (Fragment)	48	12	4	43434
173	P01868	Ig gamma-1 chain C region secreted form	52	9	1	35705
174	P01869	Ig gamma-1 chain C region, membrane-bound form	42	9	1	43387
175	P03987	Ig gamma-3 chain C region	49	15	15	43929
176	P01750	Ig heavy chain V region 102	40	4	2	12867
177	P01754	Ig heavy chain V region 1-62-3	45	3	1	12921
178	P06328	Ig heavy chain V region 1-72	45	3	1	12877
179	P01749	Ig heavy chain V region 3	61	4	2	13016
180	P18531	Ig heavy chain V region 3-6	36	3	2	13095
181	P01747	Ig heavy chain V region 36-65	24	2	1	13307
182	P18529	Ig heavy chain V region 5-76	20	3	1	12991
183	P18528	Ig heavy chain V region 6.96	36	3	1	11007
184	P18527	Ig heavy chain V region 914	53	4	1	10661
185	P01746	Ig heavy chain V region 93G7	21	2	1	15514
186	P01741	Ig heavy chain V region	11	1	1	12555
187	P01798	Ig heavy chain V-III region E109	43	4	1	12647
188	P84750	Ig kappa chain V region Mem5 (Fragment)	19	3	1	13251
189	A0A0G2JDJ8	Ig kappa chain V-I region S107A (Fragment)	13	2	1	13460
190	A0A075B5N1	Ig kappa chain V-I region S107A	13	2	1	13403
191	P01632	Ig kappa chain V-I region S107A	14	2	1	12717

192	P01631	Ig kappa chain V-II region 26-10	43	4	1	12273
193	P01629	Ig kappa chain V-II region 2S1.3	12	1	1	12221
194	P01630	Ig kappa chain V-II region 7S34.1	21	2	2	12496
195	P01626	Ig kappa chain V-II region M	21	1	1	12349
196	P01628	Ig kappa chain V-II region M	14	1	1	12496
197	P01664	Ig kappa chain V-III region CBPC 101	31	2	1	11964
198	P01656	Ig kappa chain V-III region MPC 70	26	2	1	11904
199	P01654	Ig kappa chain V-III region PC 2880/PC 1229	26	2	1	11980
200	P01660	Ig kappa chain V-III region PC 3741/TEPC 111	43	3	1	12099
201	P01670	Ig kappa chain V-III region PC 6684	44	2	1	12039
202	P01665	Ig kappa chain V-III region PC 7043	31	2	1	12002
203	P01671	Ig kappa chain V-III region PC 7175	44	2	1	12010
204	P01666	Ig kappa chain V-III region PC 7183	31	2	1	11952
205	P01668	Ig kappa chain V-III region PC 7210	31	2	1	11950
206	P01647	Ig kappa chain V-V region HP 124E1	56	5	1	11965
207	P01645	Ig kappa chain V-V region HP 93G7	56	5	1	11954
208	P01644	Ig kappa chain V-V region HP R16.7	56	5	1	11910
209	P04940	Ig kappa chain V-VI region NQ2-17.4.1	24	2	1	11561
210	P01678	Ig kappa chain V-VI region SAPC 10	56	4	3	11554
211	P01677	Ig kappa chain V-VI region TEPC 601/TEPC 191	56	4	3	11568
212	P01676	Ig kappa chain V-VI region XRPC 24	56	4	3	11584
213	P01675	Ig kappa chain V-VI region XRPC 44	56	4	3	11627
214	P01723	Ig lambda-1 chain V region	28	1	1	12222
215	P01727	Ig lambda-1 chain V region S43	26	1	1	13529
216	Q99JC1	Ig lambda-2 chain C region (Fragment)	85	6	6	11312
217	P01844	Ig lambda-2 chain C region	86	6	6	11255
218	P01728	Ig lambda-2 chain V region	28	1	1	12222
219	A0A075B6A3	Immunoglobulin heavy constant alpha (Fragment)	51	10	10	36838

220	A0A0A6YVP0	Immunoglobulin heavy constant gamma 2B (Fragment)	58	18	2	44359
221	P01867	Immunoglobulin heavy constant gamma 2B	54	16	1	44259
222	F6TQW2	Immunoglobulin heavy constant gamma 2C (Fragment)	61	15	12	44214
223	A0A075B5P5	Immunoglobulin heavy constant gamma 3 (Fragment)	59	15	15	36317
224	A0A1Y7VJN6	Immunoglobulin heavy constant gamma 3 (Fragment)	49	15	15	44018
225	A0A075B5P6	Immunoglobulin heavy constant mu (Fragment)	55	25	25	50063
226	A0A075B5R6	Immunoglobulin heavy variable 11-1 (Fragment)	50	4	4	13208
227	A0A0A6YXA5	Immunoglobulin heavy variable 1-15 (Fragment)	61	5	4	12953
228	A0A075B5U7	Immunoglobulin heavy variable 1-22 (Fragment)	56	5	3	13022
229	A0A0A6YWN3	Immunoglobulin heavy variable 13-2 (Fragment)	34	3	3	13417
230	A0A087WPN7	Immunoglobulin heavy variable 13-2	40	3	3	11343
231	A0A0A6YXT2	Immunoglobulin heavy variable 1-36 (Fragment)	13	1	1	12961
232	A0A075B5V3	Immunoglobulin heavy variable 1-36	13	1	1	12904
233	A0A075B5V5	Immunoglobulin heavy variable 1-39 (Fragment)	30	2	2	12931
234	A0A075B5R4	Immunoglobulin heavy variable 14-1 (Fragment)	38	4	1	12992
235	A0A075B5R7	Immunoglobulin heavy variable 14-2 (Fragment)	59	5	3	13002
236	A0A0A6YVR1	Immunoglobulin heavy variable 14-4 (Fragment)	21	1	1	12957
237	A0A075B5S3	Immunoglobulin heavy variable 14-4	23	1	1	12134
238	A0A0A6YY41	Immunoglobulin heavy variable 1-47 (Fragment)	53	5	5	13108
239	A0A075B5V8	Immunoglobulin heavy variable 1-47	63	5	5	11083
240	A0A075B5W1	Immunoglobulin heavy variable 1-50 (Fragment)	61	3	1	12878
241	A0A0A6YXC3	Immunoglobulin heavy variable 1-52 (Fragment)	61	4	1	13046
242	A0A075B5W2	Immunoglobulin heavy variable 1-52	61	4	1	12989
243	A0A075B5W3	Immunoglobulin heavy variable 1-53 (Fragment)	61	4	1	12792
244	A0A075B5W6	Immunoglobulin heavy variable 1-55 (Fragment)	61	3	2	12687
245	A0A075B5W9	Immunoglobulin heavy variable 1-58 (Fragment)	34	2	2	12995
246	A0A0A6YXZ4	Immunoglobulin heavy variable 1-62-2 (Fragment)	49	4	4	13359
247	A0A075B680	Immunoglobulin heavy variable 1-62-2	58	4	4	11225

248	A0A075B5X3	Immunoglobulin heavy variable 1-64 (Fragment)	61	4	1	12922
249	A0A075B5X5	Immunoglobulin heavy variable 1-66 (Fragment)	31	2	1	12922
250	A0A0G2JFL3	Immunoglobulin heavy variable 1-71 (Fragment)	49	4	4	13432
251	A0A075B5Y2	Immunoglobulin heavy variable 1-75 (Fragment)	40	3	1	12957
252	A0A0G2JFE9	Immunoglobulin heavy variable 1-76 (Fragment)	46	4	3	13021
253	A0A0B4J1N0	Immunoglobulin heavy variable 1-76	55	4	3	10879
254	A0A0G2JGS9	Immunoglobulin heavy variable 1-77 (Fragment)	35	3	2	12870
255	A0A0B4J1M0	Immunoglobulin heavy variable 1-77	42	3	2	10662
256	A0A0G2JGN3	Immunoglobulin heavy variable 1-78 (Fragment)	14	1	1	13243
257	A0A075B674	Immunoglobulin heavy variable 1-78	16	1	1	11047
258	A0A075B5Y3	Immunoglobulin heavy variable 1-80 (Fragment)	30	2	2	12763
259	A0A075B5Y4	Immunoglobulin heavy variable 1-81 (Fragment)	53	5	3	12989
260	A0A0G2JEU7	Immunoglobulin heavy variable 1-82 (Fragment)	30	3	2	12841
261	A0A0B4J1J7	Immunoglobulin heavy variable 1-82	36	3	2	10650
262	A0A075B5Y6	Immunoglobulin heavy variable 1-85 (Fragment)	26	2	2	13012
263	A0A075B5P8	Immunoglobulin heavy variable 2-2 (Fragment)	47	3	1	12578
264	A0A0A6YY69	Immunoglobulin heavy variable 2-5 (Fragment)	67	5	3	12660
265	A0A075B5Q3	Immunoglobulin heavy variable 2-5	68	5	3	12557
266	A0A075B6A7	Immunoglobulin heavy variable 2-6 (Fragment)	47	3	2	12596
267	A0A075B697	Immunoglobulin heavy variable 2-9-1 (Fragment)	37	4	3	12481
268	A0A075B5S6	Immunoglobulin heavy variable 3-1 (Fragment)	19	2	1	13109
269	A0A075B5R5	Immunoglobulin heavy variable 4-1 (Fragment)	60	6	4	12874
270	A0A0A6YWC7	Immunoglobulin heavy variable 5-15 (Fragment)	49	4	1	13000
271	A0A075B5Q9	Immunoglobulin heavy variable 5-15	49	4	1	12943
272	A0A0B4J1P4	Immunoglobulin heavy variable 5-16 (Fragment)	37	4	3	13274
273	A0A075B5R0	Immunoglobulin heavy variable 5-16	37	4	3	13217
274	A0A075B5R1	Immunoglobulin heavy variable 5-17 (Fragment)	39	4	2	12905
275	A0A075B5P9	Immunoglobulin heavy variable 5-4 (Fragment)	57	5	2	12965

276	A0A075B5Q0	Immunoglobulin heavy variable 5-6 (Fragment)	48	4	2	12863
277	A0A075B5Q2	Immunoglobulin heavy variable 5-9 (Fragment)	58	6	3	12795
278	A0A0A6YVS4	Immunoglobulin heavy variable 5-9-1 (Fragment)	40	4	2	14177
279	A0A075B5T2	Immunoglobulin heavy variable 6-3 (Fragment)	64	7	3	13259
280	A0A075B5T3	Immunoglobulin heavy variable 6-6 (Fragment)	61	6	3	13293
281	A0A075B5S2	Immunoglobulin heavy variable 7-1 (Fragment)	38	4	4	13645
282	A0A075B5R2	Immunoglobulin heavy variable 7-3 (Fragment)	44	5	4	13457
283	A0A0A6YXQ0	Immunoglobulin heavy variable 8-8 (Fragment)	36	3	2	13185
284	A0A075B5S1	Immunoglobulin heavy variable 9-1	48	3	1	11090
285	A0A075B5S9	Immunoglobulin heavy variable 9-4	43	4	2	10934
286	A0A075B5T6	Immunoglobulin heavy variable V10-3 (Fragment)	19	3	2	13545
287	A0A0A6YWI9	Immunoglobulin heavy variable V1-11 (Fragment)	19	2	2	12865
288	A0A075B5R8	Immunoglobulin heavy variable V11-2 (Fragment)	50	4	4	13167
289	A0A0A6YXJ9	Immunoglobulin heavy variable V1-12 (Fragment)	19	2	1	12873
290	A0A075B5U0	Immunoglobulin heavy variable V1-12	22	2	1	10778
291	A0A0A6YXN4	Immunoglobulin heavy variable V1-18 (Fragment)	56	5	3	12913
292	A0A0A6YWX0	Immunoglobulin heavy variable V1-19 (Fragment)	36	3	1	12914
293	A0A075B5U5	Immunoglobulin heavy variable V1-19	36	3	1	12857
294	A0A0A6YX66	Immunoglobulin heavy variable V1-20 (Fragment)	50	5	3	13128
295	A0A075B5U6	Immunoglobulin heavy variable V1-20	50	5	3	13071
296	A0A075B5V6	Immunoglobulin heavy variable V1-42	87	6	2	10776
297	A0A075B5R9	Immunoglobulin heavy variable V14-3 (Fragment)	17	2	1	12975
298	A0A0A6YXN5	Immunoglobulin heavy variable V1-43 (Fragment)	53	5	1	12983
299	A0A075B5V7	Immunoglobulin heavy variable V1-43	63	5	1	10761
300	A0A075B5T5	Immunoglobulin heavy variable V1-5	60	3	3	10846
301	A0A0A6YX72	Immunoglobulin heavy variable V15-2 (Fragment)	16	1	1	13133
302	A0A075B5T8	Immunoglobulin heavy variable V15-2	16	1	1	13032
303	A0A0A6YXE0	Immunoglobulin heavy variable V1-59 (Fragment)	61	3	1	12883

304	A0A075B5X0	Immunoglobulin heavy variable V1-59	61	3	1	12826
305	A0A0G2JFN9	Immunoglobulin heavy variable V1-67 (Fragment)	11	1	1	12947
306	A0A075B5X6	Immunoglobulin heavy variable V1-67	13	1	1	10976
307	A0A0G2JGK2	Immunoglobulin heavy variable V1-74 (Fragment)	40	4	2	12957
308	A0A075B5Y1	Immunoglobulin heavy variable V1-74	41	4	2	12900
309	A0A0A6YW37	Immunoglobulin heavy variable V1-9 (Fragment)	61	3	3	12989
310	A0A075B5T9	Immunoglobulin heavy variable V1-9	61	3	3	12932
311	A0A0G2JDE1	Immunoglobulin heavy variable V8-12 (Fragment)	36	4	3	13263
312	A0A0B4J1J5	Immunoglobulin heavy variable V9-3 (Fragment)	43	3	1	12996
313	P01592	Immunoglobulin J chain	27	4	4	18014
314	A0A0G2JEI7	Immunoglobulin kappa chain variable 12-38 (Fragment)	17	1	1	12543
315	A0A075B5M8	Immunoglobulin kappa chain variable 12-38	17	1	1	12428
316	P01635	Immunoglobulin kappa chain variable 12-41 (Fragment)	33	3	1	12581
317	A0A140T8N3	Immunoglobulin kappa chain variable 13-84 (Fragment)	19	2	1	12617
318	A0A075B666	Immunoglobulin kappa chain variable 13-85 (Fragment)	19	2	1	12709
319	A0A140T8P3	Immunoglobulin kappa chain variable 15-103 (Fragment)	14	2	1	12547
320	A0A0B4J1H6	Immunoglobulin kappa chain variable 2-137 (Fragment)	20	2	2	13131
321	A0N8I8	Immunoglobulin kappa chain variable 4-51 (Fragment)	13	1	1	12840
322	A0A075B5M2	Immunoglobulin kappa chain variable 4-61	31	2	1	10153
323	A0A0B4J1J1	Immunoglobulin kappa chain variable 5-45 (Fragment)	45	4	2	12718
324	A0A140T8N7	Immunoglobulin kappa chain variable 6-25 (Fragment)	22	3	1	12739
325	A0A140T8P5	Immunoglobulin kappa chain variable 8-24 (Fragment)	20	2	1	13264
326	A0A140T8M3	Immunoglobulin kappa chain variable 8-30 (Fragment)	30	3	1	13335
327	A0A075B5K2	Immunoglobulin kappa chain variable 9-124	47	3	3	10420
328	P01837	Immunoglobulin kappa constant	81	7	7	11934
329	A0A075B5L1	Immunoglobulin kappa variable 10-94 (Fragment)	28	1	1	12576
330	A0A140T8M0	Immunoglobulin kappa variable 1-117 (Fragment)	43	4	1	13117
331	A0A140T8M8	Immunoglobulin kappa variable 1-131 (Fragment)	9	1	1	13331

332	A0A0B4J1H9	Immunoglobulin kappa variable 1-132 (Fragment)	18	2	2	13434
333	A0A0B4J1H8	Immunoglobulin kappa variable 1-133 (Fragment)	35	5	4	13237
334	A0A140T8M2	Immunoglobulin kappa variable 12-44 (Fragment)	41	4	3	12557
335	A0A140T8P6	Immunoglobulin kappa variable 12-46 (Fragment)	41	3	2	12562
336	A0A075B5K0	Immunoglobulin kappa variable 14-126 (Fragment)	42	4	2	13058
337	A0A0G2JF45	Immunoglobulin kappa variable 14-130 (Fragment)	37	2	2	13102
338	A0A075B5J7	Immunoglobulin kappa variable 14-130	37	2	2	13045
339	A0A0B4J1I1	Immunoglobulin kappa variable 16-104 (Fragment)	23	2	2	12875
340	A0A0G2JFA8	Immunoglobulin kappa variable 17-121 (Fragment)	14	1	1	12866
341	A0A075B5K3	Immunoglobulin kappa variable 17-121	14	1	1	12692
342	A0A075B5K5	Immunoglobulin kappa variable 2-112	13	1	1	13223
343	P01627	Immunoglobulin kappa variable 2-112	13	1	1	13280
344	A0A075B5P1	Immunoglobulin kappa variable 3-1 (Fragment)	21	1	1	12972
345	A0A140T8P4	Immunoglobulin kappa variable 3-9 (Fragment)	13	2	1	12918
346	A0A075B677	Immunoglobulin kappa variable 4-53	41	2	2	10366
347	A0A075B5M4	Immunoglobulin kappa variable 4-57-1 (Fragment)	18	2	2	12723
348	A0A0G2JFU6	Immunoglobulin kappa variable 4-63 (Fragment)	7	1	1	12629
349	A0A075B5M1	Immunoglobulin kappa variable 4-63	8	1	1	10157
350	A0A075B5L7	Immunoglobulin kappa variable 4-80 (Fragment)	14	1	1	12562
351	A0A075B5L6	Immunoglobulin kappa variable 4-81	19	1	1	10234
352	A0A0B4J1I2	Immunoglobulin kappa variable 4-92 (Fragment)	9	2	1	12662
353	A0A140T8N4	Immunoglobulin kappa variable 5-37 (Fragment)	7	1	1	12606
354	A0A075B5M7	Immunoglobulin kappa variable 5-39	54	4	2	10345
355	A0A075B5N7	Immunoglobulin kappa variable 6-13	26	3	1	10441
356	A0A140T8M5	Immunoglobulin kappa variable 6-15 (Fragment)	37	4	1	12757
357	A0A140T8P2	Immunoglobulin kappa variable 6-20 (Fragment)	37	4	1	12640
358	A0A140T8N5	Immunoglobulin kappa variable 6-23 (Fragment)	22	3	1	12787
359	A0A140T8N9	Immunoglobulin kappa variable 6-32 (Fragment)	19	3	1	12656

360	A0A0G2JDG9	Immunoglobulin kappa variable 8-16 (Fragment)	40	4	2	13452
361	A0A075B5N6	Immunoglobulin kappa variable 8-16	41	4	2	13395
362	A0A140T8M4	Immunoglobulin kappa variable 8-19	53	4	2	11116
363	A0A140T8P7	Immunoglobulin kappa variable 8-21 (Fragment)	28	3	1	13128
364	A0A0G2JE47	Immunoglobulin kappa variable 8-28 (Fragment)	17	2	1	13085
365	A0A075B5N3	Immunoglobulin kappa variable 8-28	21	2	1	10903
366	A0A140T8N8	Immunoglobulin kappa variable 9-123 (Fragment)	19	2	1	12815
367	A0A075B663	Immunoglobulin lambda variable 1 (Fragment)	28	1	1	12312
368	A0A075B664	Immunoglobulin lambda variable 2	28	1	1	12165
369	A0A0B4J1K5	Immunoglobulin lambda variable 3 (Fragment)	57	5	5	13416
370	Q9DBD0	Inhibitor of carbonic anhydrase	73	40	38	76766
371	P47878	Insulin-like growth factor-binding protein 3	4	1	1	31687
372	P70389	Insulin-like growth factor-binding protein complex acid labile subunit	44	19	18	66960
373	A0A0R4J0S2	Insulin-like growth factor-binding protein complex acid labile subunit	44	19	18	66990
374	E9PVD2	Inter alpha-trypsin inhibitor, heavy chain 4	51	40	1	104588
375	A6X935	Inter alpha-trypsin inhibitor, heavy chain 4	51	40	1	104660
376	Q61702	Inter-alpha-trypsin inhibitor heavy chain H1	46	33	33	101067
377	Q61703	Inter-alpha-trypsin inhibitor heavy chain H2	36	31	31	105928
378	G3X977	Inter-alpha-trypsin inhibitor heavy chain H2	35	31	31	106361
379	Q61704	Inter-alpha-trypsin inhibitor heavy chain H3	36	23	23	99358
380	E9Q6I2	Interleukin-1 receptor accessory protein	16	10	10	73517
381	Q02257	Junction plakoglobin	3	1	1	81801
382	E9Q0F0	Keratin 78	2	3	2	112265
383	A2A513	Keratin, type I cytoskeletal 10	14	9	5	57041
384	P02535	Keratin, type I cytoskeletal 10	14	9	5	57770
385	P08730	Keratin, type I cytoskeletal 13	10	6	1	47754

386	Q61781	Keratin, type I cytoskeletal 14	22	10	2	52867
387	B1AQ77	Keratin, type I cytoskeletal 15	11	7	2	49494
388	Q61414	Keratin, type I cytoskeletal 15	11	7	2	49138
389	Q9Z2K1	Keratin, type I cytoskeletal 16	9	5	1	51606
390	Q6IFX2	Keratin, type I cytoskeletal 42	13	6	2	50133
391	Q99M73	Keratin, type II cuticular Hb4	4	3	1	64983
392	P04104	Keratin, type II cytoskeletal 1	7	7	3	65606
393	Q3TTY5	Keratin, type II cytoskeletal 2 epidermal	7	7	3	70923
394	Q3UV17	Keratin, type II cytoskeletal 2 oral	7	5	1	62845
395	Q922U2	Keratin, type II cytoskeletal 5	21	18	9	61767
396	Q3UV11	Keratin, type II cytoskeletal 6B	14	10	1	59526
397	Q9Z331	Keratin, type II cytoskeletal 6B	14	10	1	60322
398	Q6IME9	Keratin, type II cytoskeletal 72	5	3	1	56750
399	Q6NXH9	Keratin, type II cytoskeletal 73	11	8	3	58911
400	Q8VED5	Keratin, type II cytoskeletal 79	9	7	1	57552
401	P11679	Keratin, type II cytoskeletal 8	7	6	1	54565
402	Q8CCX5	Keratin-like protein KRT222	5	2	1	34198
403	A0A0R4J038	Kininogen-1	44	25	21	73101
404	Q91XL1	Leucine-rich HEV glycoprotein	24	8	8	37431
405	P42703	Leukemia inhibitory factor receptor	30	30	30	122574
406	A2AC65	Lipopolysaccharide-binding protein	9	1	1	13134
407	Q61805	Lipopolysaccharide-binding protein	2	1	1	53056
408	P06151	L-lactate dehydrogenase A chain	21	6	6	36499
409	A0A1B0GSX0	L-lactate dehydrogenase	20	6	6	39758
410	P51885	Lumican	31	8	7	38265
411	P09581	Macrophage colony-stimulating factor 1 receptor	5	4	4	109179
412	P11588	Major urinary protein 1	64	11	1	20648
413	A2CEL1	Major urinary protein 1	64	11	1	20634

414	Q58EV3	Major urinary protein 1	64	11	1	20600
415	P04938	Major urinary protein 11	64	11	1	20763
416	A2BIM8	Major urinary protein 18	64	11	1	20763
417	P11589	Major urinary protein 2	66	12	1	20664
418	P02762	Major urinary protein 6	64	11	1	20649
419	A0A571BF69	Maltase-glucoamylase	4	11	10	413024
420	P98064	Mannan-binding lectin serine protease 1	15	8	8	79968
421	Q91WP0	Mannan-binding lectin serine protease 2	10	5	5	75517
422	P39039	Mannose-binding protein A	19	5	5	25396
423	P41317	Mannose-binding protein C	22	5	5	25957
424	P45700	Mannosyl-oligosaccharide 1,2-alpha-mannosidase IA	4	2	2	73276
425	P28665	Murinoglobulin-1	64	82	44	165297
426	P04247	Myoglobin	32	3	3	17070
427	Q8VCS0	N-acetylmuramoyl-L-alanine amidase	23	9	9	57707
428	P32848	Parvalbumin alpha	33	4	4	11931
429	A0A3Q4EC30	Peptidase inhibitor 16 (Fragment)	8	1	1	15064
430	D3Z6T6	Peptidase inhibitor 16	4	1	1	29899
431	Q9ET66	Peptidase inhibitor 16	2	1	1	53650
432	P17742	Peptidyl-prolyl cis-trans isomerase A	20	3	3	17971
433	Q62009	Periostin	4	2	2	93144
434	P35700	Peroxiredoxin-1	23	4	3	22176
435	Q61171	Peroxiredoxin-2	55	10	9	21779
436	P16301	Phosphatidylcholine-sterol acyltransferase	24	8	7	49747
437	Q8VCU2	Phosphatidylinositol-glycan-specific phospholipase D	43	28	28	93624
438	P09411	Phosphoglycerate kinase 1	8	2	2	44550
439	O70250	Phosphoglycerate mutase 2	6	1	1	28827
440	P55065	Phospholipid transfer protein	8	3	3	54453
441	P97298	Pigment epithelium-derived factor	35	11	10	46234

442	P26262	Plasma kallikrein	53	29	29	71383
443	P97290	Plasma protease C1 inhibitor	39	16	16	55585
444	P20918	Plasminogen	79	52	51	90808
445	Q9Z126	Platelet factor 4	40	5	5	11243
446	O35930	Platelet glycoprotein Ib alpha chain	6	3	3	80055
447	Q9QZU3	Platelet glycoprotein V (Fragment)	3	1	1	63382
448	O08742	Platelet glycoprotein V	3	1	1	63468
449	Q60963	Platelet-activating factor acetylhydrolase	19	7	7	49258
450	Q5SX22	Polyubiquitin-B (Fragment)	13	3	3	26620
451	P0CG49	Polyubiquitin-B	10	3	3	34369
452	E9Q5F6	Polyubiquitin-C (Fragment)	15	3	3	22592
453	P0CG50	Polyubiquitin-C	4	3	3	82550
454	A0A0G2JET4	Predicted gene 42543 (Fragment)	26	2	2	13114
455	A0A0G2JGT0	Predicted gene 43218 (Fragment)	43	4	2	12726
456	A0A0N4SVU1	Predicted gene 7298	22	26	1	165287
457	A0A075B5J6	Predicted gene, 20730	11	1	1	13072
458	A0A2R8VHP3	Predicted pseudogene 5478	7	5	1	57920
459	A0A0A6YW67	Predicted pseudogene 8797	40	3	3	8728
460	Q61838	Pregnancy zone protein	70	88	86	165852
461	Q5SX49	Profilin	27	2	2	11820
462	P62962	Profilin-1	21	2	2	14957
463	P11680	Properdin	30	10	9	50327
464	Q8BKE0	Proteasome subunit alpha type-2 (Fragment)	10	1	1	20787
465	P49722	Proteasome subunit alpha type-2	8	1	1	25927
466	Q9R1P0	Proteasome subunit alpha type-4	7	2	2	29471
467	Q07456	Protein AMBP	27	9	9	39029
468	Q8R121	Protein Z-dependent protease inhibitor	41	13	13	51797
469	P19221	Prothrombin	52	37	35	70269

470	Q543K9	Purine nucleoside phosphorylase	7	2	2	32263
471	P23492	Purine nucleoside phosphorylase	7	2	2	32277
472	P52480	Pyruvate kinase PKM	9	4	4	57845
473	Q9WVF5	Receptor protein-tyrosine kinase	30	16	16	72907
474	H7BWY6	Retinol-binding protein 4	36	9	9	28405
475	Q00724	Retinol-binding protein 4	44	9	9	23206
476	P70274	Selenoprotein P	24	9	8	42706
477	A0A0R4J0I1	Serine protease inhibitor A3K	44	18	2	46673
478	P07759	Serine protease inhibitor A3K	63	27	7	46880
479	Q03734	Serine protease inhibitor A3M	44	18	6	47064
480	Q91WP6	Serine protease inhibitor A3N	43	16	11	46718
481	Q921I1	Serotransferrin	78	66	64	76724
482	P31532	Serum amyloid A-4 protein	51	7	7	15088
483	P12246	Serum amyloid P-component	57	10	10	26247
484	P52430	Serum paraoxonase/arylesterase 1	57	18	18	39565
485	Q8BND5	Sulfhydryl oxidase 1	30	19	19	82785
486	P08228	Superoxide dismutase [Cu-Zn]	27	4	4	15943
487	P43025	Tetranectin	20	3	3	22257
488	P10639	Thioredoxin	9	1	1	11675
489	P35441	Thrombospondin-1	29	26	26	129647
490	Q80YQ1	Thrombospondin-1	29	26	26	129690
491	Q9Z1T2	Thrombospondin-4	4	3	3	106366
492	E9PZA7	Transformation/transcription domain-associated protein	0	1	1	434049
493	E9QLK7	Transformation/transcription domain-associated protein	0	1	1	435804
494	P07309	Transthyretin	44	5	5	15776
495	A6XA75	Trem-like transcript 1 protein	14	2	2	26101
496	Q8K558	Trem-like transcript 1 protein	10	2	2	33522
497	P17751	Triosephosphate isomerase	20	4	4	26713

498	Q7TMM9	Tubulin beta-2A chain	3	1	1	49907
499	Q9CWF2	Tubulin beta-2B chain	3	1	1	49953
500	Q9D6F9	Tubulin beta-4A chain	3	1	1	49586
501	P68372	Tubulin beta-4B chain	3	1	1	49831
502	P99024	Apolipoprotein C-II E3	3	1	1	49671
503	P62983	Ubiquitin-40S ribosomal protein S27a	20	3	3	17951
504	E9Q9J0	Ubiquitin-60S ribosomal protein L40 (Fragment)	32	3	3	10914
505	P62984	Ubiquitin-60S ribosomal protein L40	24	3	3	14728
506	Q68FD9	UPF0606 protein KIAA1549	1	2	2	209219
507	P29533	Vascular cell adhesion protein 1	6	3	3	81317
508	Q3UPN1	Vascular cell adhesion protein 1	6	3	3	81317
509	Q9QZ25	Vascular non-inflammatory molecule 3	16	6	6	56305
510	P21614	Vitamin D-binding protein	71	31	31	53600
511	P33587	Vitamin K-dependent protein C	5	2	2	51818
512	Q08761	Vitamin K-dependent protein S	2	2	2	74934
513	Q9CQW3	Vitamin K-dependent protein Z	12	4	4	44304
514	P29788	Vitronectin	40	15	15	54849
515	Q8CIZ8	von Willebrand factor	0	1	1	309266
516	E9QPU1	von Willebrand factor	0	1	1	309154
517	Q64726	Zinc-alpha-2-glycoprotein	50	15	14	35332

Table S2. Complete list of proteins identified in the chronic phase

N°	Access	Description	Coverage (%)	Identified Peptides	Unique Peptides	Molecular Mass (Da)
1	P60710	Actin, cytoplasmic 1	29	10	1	41737
2	P63260	Actin, cytoplasmic 2	29	10	1	41793
3	Q60994	Adiponectin	15	3	3	26809
4	O89020	Afamin	47	31	31	69379
5	P07724	Albumin	86	62	62	68693
6	Q91VB8	Alpha globin 1	84	8	8	15112
7	Q60590	Alpha-1-acid glycoprotein 1	18	5	5	23895
8	P22599	Alpha-1-antitrypsin 1-2	53	20	6	45975
9	Q00897	Alpha-1-antitrypsin 1-4	57	26	7	45998
10	Q00898	Alpha-1-antitrypsin 1-5	24	12	1	45891
11	Q19LI2	Alpha-1B-glycoprotein	53	24	23	56554
12	Q61247	Alpha-2-antiplasmin	36	18	17	54972
13	P29699	Alpha-2-HS-glycoprotein	44	10	10	37326
14	P00687	Alpha-amylase 1	11	4	4	57644
15	Q9CY02	Alpha-hemoglobin-stabilizing protein	12	2	2	11832
16	A0A5F8MPW1	Angiotensinogen	26	8	8	52030
17	Q3UTR7	Angiotensinogen	25	8	8	52670
18	P32261	Antithrombin-III	54	25	23	52004
19	Q00623	Apolipoprotein A-I	75	25	25	30616
20	P09813	Apolipoprotein A-II	46	4	4	11309
21	P06728	Apolipoprotein A-IV	59	24	23	45029
22	E9Q414	Apolipoprotein B-100	18	64	64	509437
23	P34928	Apolipoprotein C-I	39	5	5	9696
24	Q05020	Apolipoprotein C-II	22	1	1	10741
25	P33622	Apolipoprotein C-III	38	2	2	10982
26	A0A0R4J1N3	Apolipoprotein C-III	38	2	2	10922

27	E9QP56	Apolipoprotein C-III	28	2	2	15163
28	Q61268	Apolipoprotein C-IV	6	1	1	14288
29	P51910	Apolipoprotein D	24	4	4	21530
30	P08226	Apolipoprotein E	46	16	16	35867
31	Q9Z1R3	Apolipoprotein M	18	4	4	21273
32	G3X9D6	Apolipoprotein N	3	1	1	27965
33	Q01339	Beta-2-glycoprotein 1	55	18	18	38619
34	P01887	Beta-2-microglobulin	32	3	3	13779
35	A8DUK4	Beta-globin	95	17	5	15748
36	P15327	Bisphosphoglycerate mutase	10	2	2	29978
37	P07743	BPI fold-containing family A member 2	24	5	5	24753
38	B7ZCG3	BPI fold-containing family A member 2 (Fragment)	27	5	5	22321
39	P08607	C4b-binding protein	28	10	10	51524
40	E9Q3M9	Capping protein-inhibiting regulator of actin-like	1	2	1	125910
41	P13634	Carbonic anhydrase 1	40	7	7	28331
42	P00920	Carbonic anhydrase 2	47	10	10	29033
43	P16015	Carbonic anhydrase 3	23	5	5	29366
44	P23953	Carboxylesterase 1C	46	22	13	61056
45	Q8VCT4	Carboxylesterase 1D	10	4	1	61788
46	Q64176	Carboxylesterase 1E	4	2	1	61582
47	D3Z5G7	Carboxylic ester hydrolase	31	16	8	62197
48	H3BL34	Carboxylic ester hydrolase	4	2	1	61511
49	Q9JHH6	Carboxypeptidase B2	10	5	5	48871
50	Q9JJN5	Carboxypeptidase N catalytic chain	31	11	11	51845
51	Q9DBB9	Carboxypeptidase N subunit 2	27	13	13	60479
52	Q9WVJ3	Carboxypeptidase Q	8	3	3	51813
53	P10605	Cathepsin B	5	1	1	37280
54	Q9QWK4	CD5 antigen-like	48	18	18	38863
55	Q6A078	Centrosomal protein of 290 kDa	0	1	1	289075
56	E9Q9M0	Centrosomal protein of 290 kDa	0	1	1	289972
57	Q61147	Ceruloplasmin	44	45	44	121151

58	E9PZD8	Ceruloplasmin	43	45	44	124211
59	Q9EQI5	Chemokine subfamily B Cys-X-Cys	41	2	2	12252
60	Q03311	Cholinesterase	10	4	4	68462
61	P05208	Chymotrypsin-like elastase family member 2A	8	1	1	28914
62	Q06890	Clusterin	34	17	10	51656
63	E9Q2G2	Clusterin (Fragment)	18	8	1	23651
64	O88783	Coagulation factor V	2	3	3	247228
65	O88947	Coagulation factor X	21	10	10	54018
66	Q3U3V1	Coagulation factor X	21	10	10	55255
67	Q80YC5	Coagulation factor XII	20	10	10	65701
68	Q07968	Coagulation factor XIII B chain	13	8	8	76195
69	P98086	Complement C1q subcomponent subunit A	20	3	3	25974
70	P14106	Complement C1q subcomponent subunit B	36	7	7	26717
71	Q02105	Complement C1q subcomponent subunit C	20	4	4	25992
72	Q8CG16	Complement C1r-A subcomponent	21	10	2	80073
73	Q8CG14	Complement C1s-1 subcomponent	14	7	4	76858
74	E9Q6C2	Complement C1s-1 subcomponent	14	7	4	77500
75	B8JJN2	Complement C2	8	4	2	69685
76	P21180	Complement C2	6	4	2	84742
77	P01027	Complement C3	62	94	88	186483
78	P01029	Complement C4-B	31	46	46	192914
79	P06684	Complement C5	19	24	24	188877
80	A2A998	Complement component C8 alpha chain	10	5	5	61008
81	A2A997	Complement component C8 alpha chain	10	5	5	65393
82	Q8K182	Complement component C8 alpha chain	10	5	5	66080
83	Q8BH35	Complement component C8 beta chain	12	5	5	66229
84	Q9DAC2	Complement component C8 gamma chain	28	4	4	18946
85	Q8VCG4	Complement component C8 gamma chain	23	4	4	22508
86	P06683	Complement component C9	25	14	14	62002
87	A0A0R4J032	Complement component C9	25	14	14	63426
88	B8JJN0	Complement factor B	27	34	32	142325

89	P03953	Complement factor D	23	4	4	28057
90	P06909	Complement factor H	50	48	37	139137
91	E9Q8I0	Complement factor H	49	48	37	141271
92	Q61406	Complement factor H-related 1	9	3	2	38443
93	Q4LDF6	Complement factor H-related 2	38	10	1	37932
94	E9PUM5	Complement factor H-related 4	13	10	5	91445
95	E9Q8B5	Complement factor H-related 4	12	10	5	99438
96	Q61129	Complement factor I	42	20	19	67261
97	Q06770	Corticosteroid-binding globulin	39	15	15	44769
98	P14847	C-reactive protein	28	4	4	25360
99	P07310	Creatine kinase M-type	16	5	5	43045
100	Q8CFZ6	C-type lectin domain family 3, member b	7	1	1	22255
101	Q8BPB5	EGF-containing fibulin-like extracellular matrix protein 1	5	2	2	54953
102	Q01279	Epidermal growth factor receptor	5	5	5	134853
103	A0A2I3BRQ1	Expressed sequence AI182371	12	4	4	40054
104	F8WI14	Extracellular matrix protein 1	11	5	5	62718
105	Q61508	Extracellular matrix protein 1	11	5	5	62832
106	Q9QXC1	Fetuin-B	47	12	12	42713
107	E9PV24	Fibrinogen alpha chain	3	1	1	87429
108	B7ZNJ1	Fibronectin	43	63	63	239720
109	B9EHT6	Fibronectin	41	63	63	250295
110	A0A087WSN6	Fibronectin	41	63	63	253009
111	Q08879	Fibulin-1	3	2	2	78033
112	Q923D2	Flavin reductase (NADPH)	28	4	4	22197
113	E9PZC3	Flavin reductase (NADPH)	23	4	4	26647
114	P13020	Gelsolin	35	25	24	85942
115	A0A1C7ZMZ5	Glutathione peroxidase (Fragment)	29	5	5	22305
116	P46412	Glutathione peroxidase 3	25	5	5	25424
117	A0A1C7ZMZ7	Glutathione peroxidase 3 (Fragment)	26	5	5	23976
118	P10649	Glutathione S-transferase Mu 1	6	1	1	25970
119	F6WHQ7	Glutathione S-transferase Mu 1 (Fragment)	6	1	1	22587

120	A2AE89	Glutathione transferase	5	1	1	28556
121	P16858	Glyceraldehyde-3-phosphate dehydrogenase	16	4	4	35810
122	A0A0A0MQF6	Glyceraldehyde-3-phosphate dehydrogenase	15	4	4	38653
123	S4R257	Glyceraldehyde-3-phosphate dehydrogenase (Fragment)	19	4	4	29939
124	E9QAH1	Golgi autoantigen, golgin subfamily b, macrogolgin 1	0	2	2	365297
125	E9PVZ8	Golgi autoantigen, golgin subfamily b, macrogolgin 1	0	2	2	370154
126	P01902	H-2 class I histocompatibility antigen, K-D alpha chain	8	3	1	41490
127	P01898	H-2 class I histocompatibility antigen, Q10 alpha chain	38	11	7	37251
128	Q61646	Haptoglobin	46	15	15	38752
129	Q504P4	Heat shock cognate 71 kDa protein	2	1	1	68779
130	P63017	Heat shock cognate 71 kDa protein	2	1	1	70871
131	P02088	Hemoglobin subunit beta-1	95	17	2	15840
132	P02089	Hemoglobin subunit beta-2	95	18	9	15878
133	Q91X72	Hemopexin	63	30	29	51318
134	P49182	Heparin cofactor 2	28	13	13	54497
135	Q9R098	Hepatocyte growth factor activator	6	3	3	70568
136	Q9ESB3	Histidine-rich glycoprotein	29	15	15	59163
137	A0A0R4J039	Histidine-rich glycoprotein	29	15	15	60439
138	Q8HWB2	Histocompatibility 2, Q region locus 4	21	7	1	39618
139	Q3V1J8	Hyaluronan-binding protein 2	5	3	3	57326
140	E9Q092	Hyaluronan-binding protein 2	4	3	3	61298
141	E9QM92	Hyaluronan-binding protein 2	4	3	3	61823
142	Q8K0D2	Hyaluronan-binding protein 2	4	3	3	62357
143	A0A075B5P4	Ig gamma-1 chain C region secreted form (Fragment)	58	12	12	35752
144	A0A0A6YWR2	Ig gamma-1 chain C region secreted form (Fragment)	48	12	12	43434
145	P01863	Ig gamma-2A chain C region, A allele	21	4	1	36389
146	P01865	Ig gamma-2A chain C region, membrane-bound form	17	4	1	43949
147	P03987	Ig gamma-3 chain C region	57	16	16	43929
148	P01741	Ig heavy chain V region	11	1	1	12555
149	P01750	Ig heavy chain V region 102	40	4	2	12867
150	P01748	Ig heavy chain V region 23	19	2	1	12772

151	P18531	Ig heavy chain V region 3-6	19	2	2	13095
152	P01747	Ig heavy chain V region 36-65	20	2	1	13307
153	P18528	Ig heavy chain V region 6.96	36	3	1	11007
154	P18527	Ig heavy chain V region 914	36	4	2	10661
155	P01746	Ig heavy chain V region 93G7	17	2	1	15514
156	P06330	Ig heavy chain V region AC38 205.12	72	6	1	12934
157	P01810	Ig heavy chain V region J539	19	2	1	13240
158	P03980	Ig heavy chain V region TEPC 1017	16	2	1	15576
159	P01798	Ig heavy chain V-III region E109	27	3	1	12647
160	P84750	Ig kappa chain V region Mem5 (Fragment)	31	5	2	13251
161	P01632	Ig kappa chain V-I region S107A	18	3	1	12717
162	P01631	Ig kappa chain V-II region 26-10	48	5	1	12273
163	P01629	Ig kappa chain V-II region 2S1.3	12	1	1	12221
164	P01630	Ig kappa chain V-II region 7S34.1	26	3	2	12496
165	P01628	Ig kappa chain V-II region M	33	4	3	12496
166	P01626	Ig kappa chain V-II region M	21	1	1	12349
167	P01664	Ig kappa chain V-III region CBPC 101	31	2	1	11964
168	P01660	Ig kappa chain V-III region PC 3741/TEPC 111	31	3	1	12099
169	P01667	Ig kappa chain V-III region PC 6308	31	2	1	12071
170	P01665	Ig kappa chain V-III region PC 7043	31	2	1	12002
171	P01666	Ig kappa chain V-III region PC 7183	31	2	1	11952
172	P01668	Ig kappa chain V-III region PC 7210	31	2	1	11950
173	P01672	Ig kappa chain V-III region PC 7940	41	3	1	12039
174	P01645	Ig kappa chain V-V region HP 93G7	61	6	1	11954
175	P01644	Ig kappa chain V-V region HP R16.7	61	6	1	11910
176	P01636	Ig kappa chain V-V region M	34	3	1	12030
177	P01649	Ig kappa chain V-V regions	22	4	1	12057
178	P01679	Ig kappa chain V-VI region J539	29	3	2	11502
179	P01678	Ig kappa chain V-VI region SAPC 10	29	3	2	11554
180	P01677	Ig kappa chain V-VI region TEPC 601/TEPC 191	29	3	2	11568
181	P01676	Ig kappa chain V-VI region XRPC 24	29	3	2	11584

182	P01675	Ig kappa chain V-VI region XRPC 44	29	3	2	11627
183	P01844	Ig lambda-2 chain C region	71	4	4	11255
184	Q99JC1	Ig lambda-2 chain C region (Fragment)	70	4	4	11312
185	P01728	Ig lambda-2 chain V region	14	2	2	12222
186	P01729	Ig lambda-2 chain V region M	12	2	2	13418
187	A0A075B6A3	Immunoglobulin heavy constant alpha (Fragment)	40	8	8	36838
188	A0A0A6YXW6	Immunoglobulin heavy constant alpha (Fragment)	35	8	8	42078
189	P01867	Immunoglobulin heavy constant gamma 2B	51	14	1	44259
190	A0A0A6YVP0	Immunoglobulin heavy constant gamma 2B (Fragment)	55	16	2	44359
191	F6TQW2	Immunoglobulin heavy constant gamma 2C (Fragment)	59	14	12	44214
192	A0A1Y7VJN6	Immunoglobulin heavy constant gamma 3 (Fragment)	56	16	16	44018
193	A0A075B5P6	Immunoglobulin heavy constant mu (Fragment)	62	26	26	50063
194	A0A075B5R6	Immunoglobulin heavy variable 11-1 (Fragment)	23	2	2	13208
195	A0A0A6YXA5	Immunoglobulin heavy variable 1-15 (Fragment)	61	5	4	12953
196	A0A075B5U7	Immunoglobulin heavy variable 1-22 (Fragment)	39	5	2	13022
197	A0A087WPN7	Immunoglobulin heavy variable 13-2	27	2	2	11343
198	A0A0A6YWN3	Immunoglobulin heavy variable 13-2 (Fragment)	23	2	2	13417
199	A0A075B5R4	Immunoglobulin heavy variable 14-1 (Fragment)	31	3	1	12992
200	A0A075B5R7	Immunoglobulin heavy variable 14-2 (Fragment)	26	3	2	13002
201	A0A075B5V8	Immunoglobulin heavy variable 1-47	48	4	4	11083
202	A0A0A6YY41	Immunoglobulin heavy variable 1-47 (Fragment)	40	4	4	13108
203	A0A075B5W2	Immunoglobulin heavy variable 1-52	41	3	1	12989
204	A0A0A6YXC3	Immunoglobulin heavy variable 1-52 (Fragment)	40	3	1	13046
205	A0A075B5W3	Immunoglobulin heavy variable 1-53 (Fragment)	40	3	1	12792
206	A0A075B5W6	Immunoglobulin heavy variable 1-55 (Fragment)	34	2	2	12687
207	A0A075B5W9	Immunoglobulin heavy variable 1-58 (Fragment)	13	1	1	12995
208	A0A075B680	Immunoglobulin heavy variable 1-62-2	47	3	3	11225
209	A0A0A6YXZ4	Immunoglobulin heavy variable 1-62-2 (Fragment)	39	3	3	13359
210	A0A075B5X7	Immunoglobulin heavy variable 1-69	41	3	2	12892
211	A0A0G2JFS5	Immunoglobulin heavy variable 1-69 (Fragment)	40	3	2	12949
212	A0A0G2JFL3	Immunoglobulin heavy variable 1-71 (Fragment)	39	3	3	13432

213	A0A075B5Y2	Immunoglobulin heavy variable 1-75 (Fragment)	40	3	1	12957
214	A0A0B4J1N0	Immunoglobulin heavy variable 1-76	87	6	5	10879
215	A0A0G2JFE9	Immunoglobulin heavy variable 1-76 (Fragment)	73	6	5	13021
216	A0A0B4J1M0	Immunoglobulin heavy variable 1-77	60	5	4	10662
217	A0A075B674	Immunoglobulin heavy variable 1-78	16	1	1	11047
218	A0A0G2JGN3	Immunoglobulin heavy variable 1-78 (Fragment)	14	1	1	13243
219	A0A075B5Y4	Immunoglobulin heavy variable 1-81 (Fragment)	53	5	3	12989
220	A0A0B4J1J7	Immunoglobulin heavy variable 1-82	20	2	1	10650
221	A0A0G2JEU7	Immunoglobulin heavy variable 1-82 (Fragment)	17	2	1	12841
222	A0A075B5Y6	Immunoglobulin heavy variable 1-85 (Fragment)	26	2	2	13012
223	A0A075B5P8	Immunoglobulin heavy variable 2-2 (Fragment)	19	2	1	12578
224	A0A075B5Q3	Immunoglobulin heavy variable 2-5	28	3	2	12557
225	A0A0A6YY69	Immunoglobulin heavy variable 2-5 (Fragment)	28	3	2	12660
226	A0A075B697	Immunoglobulin heavy variable 2-9-1 (Fragment)	19	3	3	12481
227	A0A075B5R5	Immunoglobulin heavy variable 4-1 (Fragment)	53	4	3	12874
228	A0A075B5Q4	Immunoglobulin heavy variable 5-12 (Fragment)	27	3	1	13075
229	A0A075B5R0	Immunoglobulin heavy variable 5-16	9	1	1	13217
230	A0A0B4J1P4	Immunoglobulin heavy variable 5-16 (Fragment)	9	1	1	13274
231	A0A075B5R1	Immunoglobulin heavy variable 5-17 (Fragment)	23	3	1	12905
232	A0A075B5P9	Immunoglobulin heavy variable 5-4 (Fragment)	23	3	1	12965
233	A0A075B5Q6	Immunoglobulin heavy variable 5-9-1	18	2	1	13067
234	A0A0A6YVS4	Immunoglobulin heavy variable 5-9-1 (Fragment)	17	2	1	14177
235	A0A075B5T2	Immunoglobulin heavy variable 6-3 (Fragment)	38	5	3	13259
236	A0A075B5T3	Immunoglobulin heavy variable 6-6 (Fragment)	45	5	3	13293
237	A0A075B5S2	Immunoglobulin heavy variable 7-1 (Fragment)	20	2	1	13645
238	A0A075B5R3	Immunoglobulin heavy variable 7-2	21	3	1	13351
239	A0A0A6YX91	Immunoglobulin heavy variable 7-2 (Fragment)	21	3	1	13435
240	A0A075B5R2	Immunoglobulin heavy variable 7-3 (Fragment)	32	4	2	13457
241	A0A075B5S5	Immunoglobulin heavy variable 7-4	12	2	1	13453
242	A0A0A6YXL5	Immunoglobulin heavy variable 7-4 (Fragment)	12	2	1	13537
243	A0A0A6YXQ0	Immunoglobulin heavy variable 8-8 (Fragment)	18	2	1	13185

244	A0A075B5S1	Immunoglobulin heavy variable 9-1	44	3	1	11090
245	A0A075B5T6	Immunoglobulin heavy variable V10-3 (Fragment)	21	3	2	13545
246	A0A075B5R8	Immunoglobulin heavy variable V11-2 (Fragment)	23	2	2	13167
247	A0A0A6YXN4	Immunoglobulin heavy variable V1-18 (Fragment)	44	5	3	12913
248	A0A075B5U6	Immunoglobulin heavy variable V1-20	24	3	1	13071
249	A0A0A6YX66	Immunoglobulin heavy variable V1-20 (Fragment)	24	3	1	13128
250	A0A075B5V6	Immunoglobulin heavy variable V1-42	71	6	1	10776
251	A0A075B5V7	Immunoglobulin heavy variable V1-43	48	5	1	10761
252	A0A0A6YXN5	Immunoglobulin heavy variable V1-43 (Fragment)	40	5	1	12983
253	A0A075B5T5	Immunoglobulin heavy variable V1-5	45	2	2	10846
254	A0A075B5X6	Immunoglobulin heavy variable V1-67	24	2	2	10976
255	A0A0G2JFN9	Immunoglobulin heavy variable V1-67 (Fragment)	21	2	2	12947
256	A0A075B5T7	Immunoglobulin heavy variable V1-7 (Fragment)	31	2	1	13160
257	A0A075B5Y1	Immunoglobulin heavy variable V1-74	41	4	2	12900
258	A0A0G2JGK2	Immunoglobulin heavy variable V1-74 (Fragment)	40	4	2	12957
259	A0A075B5T9	Immunoglobulin heavy variable V1-9	22	1	1	12932
260	A0A0A6YW37	Immunoglobulin heavy variable V1-9 (Fragment)	21	1	1	12989
261	A0A0G2JDE1	Immunoglobulin heavy variable V8-12 (Fragment)	24	3	2	13263
262	P01592	Immunoglobulin J chain	21	3	3	18014
263	A0A075B5M8	Immunoglobulin kappa chain variable 12-38	17	1	1	12428
264	A0A0G2JEI7	Immunoglobulin kappa chain variable 12-38 (Fragment)	17	1	1	12543
265	P01635	Immunoglobulin kappa chain variable 12-41 (Fragment)	28	4	2	12581
266	A0A140T8N3	Immunoglobulin kappa chain variable 13-84 (Fragment)	19	2	1	12617
267	A0A075B666	Immunoglobulin kappa chain variable 13-85 (Fragment)	19	2	1	12709
268	A0A140T8P3	Immunoglobulin kappa chain variable 15-103 (Fragment)	14	2	1	12547
269	A0A0G2JDU3	Immunoglobulin kappa chain variable 20-101-2 (Fragment)	16	2	1	12629
270	A0N8I8	Immunoglobulin kappa chain variable 4-51 (Fragment)	13	1	1	12840
271	J3QMZ0	Immunoglobulin kappa chain variable 4-54	19	1	1	10236
272	A0A075B5M2	Immunoglobulin kappa chain variable 4-61	31	2	1	10153
273	A0A0B4J1J2	Immunoglobulin kappa chain variable 5-43 (Fragment)	23	2	1	12600
274	A0A0B4J1J1	Immunoglobulin kappa chain variable 5-45 (Fragment)	45	4	2	12718

275	A0A140T8N7	Immunoglobulin kappa chain variable 6-25 (Fragment)	11	2	1	12739
276	A0A140T8P5	Immunoglobulin kappa chain variable 8-24 (Fragment)	30	3	2	13264
277	A0A140T8M3	Immunoglobulin kappa chain variable 8-30 (Fragment)	22	2	1	13335
278	P01639	Immunoglobulin kappa chain variable 9-120	19	3	1	14311
279	A0A075B5K2	Immunoglobulin kappa chain variable 9-124	47	3	2	10420
280	P01837	Immunoglobulin kappa constant	88	8	8	11934
281	A0A075B5L1	Immunoglobulin kappa variable 10-94 (Fragment)	28	1	1	12576
282	A0A140T8M0	Immunoglobulin kappa variable 1-117 (Fragment)	43	4	1	13117
283	A0A140T8M8	Immunoglobulin kappa variable 1-131 (Fragment)	9	1	1	13331
284	A0A0B4J1H9	Immunoglobulin kappa variable 1-132 (Fragment)	27	3	2	13434
285	A0A0B4J1H8	Immunoglobulin kappa variable 1-133 (Fragment)	35	6	1	13237
286	A0A0B4J1H7	Immunoglobulin kappa variable 1-135 (Fragment)	35	5	1	13305
287	A0A140T8M2	Immunoglobulin kappa variable 12-44 (Fragment)	28	3	1	12557
288	A0A140T8P6	Immunoglobulin kappa variable 12-46 (Fragment)	41	3	2	12562
289	A0A075B5K0	Immunoglobulin kappa variable 14-126 (Fragment)	29	3	2	13058
290	A0A075B5J7	Immunoglobulin kappa variable 14-130	11	1	1	13045
291	A0A0G2JF45	Immunoglobulin kappa variable 14-130 (Fragment)	11	1	1	13102
292	A0A0B4J1I1	Immunoglobulin kappa variable 16-104 (Fragment)	23	2	2	12875
293	A0A075B5K3	Immunoglobulin kappa variable 17-121	14	1	1	12692
294	A0A0G2JFA8	Immunoglobulin kappa variable 17-121 (Fragment)	14	1	1	12866
295	A0A075B5J9	Immunoglobulin kappa variable 17-127	14	1	1	12330
296	A0A0G2JDN5	Immunoglobulin kappa variable 17-127 (Fragment)	14	1	1	12759
297	A0A140T8P4	Immunoglobulin kappa variable 3-9 (Fragment)	13	2	1	12918
298	A0A075B677	Immunoglobulin kappa variable 4-53	23	1	1	10366
299	A0A0G2JFC6	Immunoglobulin kappa variable 4-53 (Fragment)	18	1	1	12820
300	A0A075B5M1	Immunoglobulin kappa variable 4-63	17	2	2	10157
301	A0A0G2JFU6	Immunoglobulin kappa variable 4-63 (Fragment)	14	2	2	12629
302	A0A075B5L7	Immunoglobulin kappa variable 4-80 (Fragment)	14	1	1	12562
303	A0A075B5M7	Immunoglobulin kappa variable 5-39	35	3	1	10345
304	A0A0G2JDV4	Immunoglobulin kappa variable 5-39 (Fragment)	29	3	1	12541
305	A0A075B5N7	Immunoglobulin kappa variable 6-13	33	4	2	10441

306	A0A140T8P1	Immunoglobulin kappa variable 6-14 (Fragment)	11	2	1	12848
307	A0A140T8M5	Immunoglobulin kappa variable 6-15 (Fragment)	30	4	2	12757
308	A0A140T8P2	Immunoglobulin kappa variable 6-20 (Fragment)	37	4	1	12640
309	A0A140T8N5	Immunoglobulin kappa variable 6-23 (Fragment)	11	2	1	12787
310	A0A140T8N9	Immunoglobulin kappa variable 6-32 (Fragment)	19	3	1	12656
311	A0A0B4J1J3	Immunoglobulin kappa variable 8-18 (Fragment)	7	1	1	13764
312	A0A140T8M4	Immunoglobulin kappa variable 8-19	45	3	2	11116
313	A0A140T8P7	Immunoglobulin kappa variable 8-21 (Fragment)	21	3	2	13128
314	A0A075B5N5	Immunoglobulin kappa variable 8-26 (Fragment)	7	1	1	13821
315	A0A075B5N3	Immunoglobulin kappa variable 8-28	21	2	1	10903
316	A0A0G2JE47	Immunoglobulin kappa variable 8-28 (Fragment)	17	2	1	13085
317	A0A075B664	Immunoglobulin lambda variable 2	14	2	2	12165
318	D3YY36	Inhibitor of carbonic anhydrase	46	26	24	68601
319	Q9DBD0	Inhibitor of carbonic anhydrase	41	26	24	76766
320	P47878	Ig kappa chain V-V regions growth factor-binding protein 3	4	1	1	31687
321	P70389	Insulin-like growth factor-binding protein complex acid labile subunit	34	14	14	66960
322	A0A0R4J0S2	Insulin-like growth factor-binding protein complex acid labile subunit	34	14	14	66990
323	E9Q5L2	Inter alpha-trypsin inhibitor, heavy chain 4	43	32	32	102823
324	E9PVD2	Inter alpha-trypsin inhibitor, heavy chain 4	43	32	32	104588
325	A6X935	Inter alpha-trypsin inhibitor, heavy chain 4	42	32	32	104660
326	Q61702	Inter-alpha-trypsin inhibitor heavy chain H1	34	26	26	101067
327	Q61703	Inter-alpha-trypsin inhibitor heavy chain H2	31	27	27	105928
328	G3X977	Inter-alpha-trypsin inhibitor heavy chain H2	31	27	27	106361
329	Q61704	Inter-alpha-trypsin inhibitor heavy chain H3	28	19	19	99358
330	O35664	Interferon alpha/beta receptor 2	4	2	2	56578
331	E9Q6I2	Interleukin-1 receptor accessory protein	14	9	9	73517
332	E9Q0F0	Keratin 78	2	2	1	112265
333	A2A513	Keratin, type I cytoskeletal 10	11	6	3	57041
334	P02535	Keratin, type I cytoskeletal 10	11	6	3	57770

335	Q9QWL7	Keratin, type I cytoskeletal 17	10	5	1	48162
336	P19001	Keratin, type I cytoskeletal 19	10	4	1	44542
337	Q6IFX2	Keratin, type I cytoskeletal 42	5	3	1	50133
338	P04104	Keratin, type II cytoskeletal 1	7	6	3	65606
339	Q3UV17	Keratin, type II cytoskeletal 2 oral	4	3	1	62845
340	Q922U2	Keratin, type II cytoskeletal 5	9	8	3	61767
341	Q3UV11	Keratin, type II cytoskeletal 6B	8	5	1	59526
342	Q9Z331	Keratin, type II cytoskeletal 6B	8	5	1	60322
343	Q6IME9	Keratin, type II cytoskeletal 72	5	2	1	56750
344	Q8VED5	Keratin, type II cytoskeletal 79	7	5	1	57552
345	A0A0R4J038	Kininogen-1	40	22	18	73101
346	E9Q4P0	KxDL motif-containing protein 1 (Fragment)	26	4	4	22234
347	Q91XL1	Leucine-rich HEV glycoprotein	11	4	4	37431
348	P42703	Leukemia inhibitory factor receptor	17	15	15	122574
349	A0A1B0GSR9	L-lactate dehydrogenase	12	4	4	34599
350	A0A1B0GSX0	L-lactate dehydrogenase	10	4	4	39758
351	P06151	L-lactate dehydrogenase A chain	11	4	4	36499
352	P51885	Lumican	24	7	7	38265
353	P08905	Lysozyme C-2	22	2	2	16689
354	P09581	Macrophage colony-stimulating factor 1 receptor	3	3	3	109179
355	A2CEL1	Major urinary protein 1	37	7	1	20634
356	P11588	Major urinary protein 1	37	7	1	20648
357	P04938	Major urinary protein 11	36	7	1	20763
358	A2BIM8	Major urinary protein 18	36	7	1	20763
359	P11589	Major urinary protein 2	39	8	2	20664
360	P02762	Major urinary protein 6	37	7	1	20649
361	A0A571BF69	Maltase-glucoamylase	5	14	14	413024
362	P98064	Mannan-binding lectin serine protease 1	9	4	4	79968
363	P39039	Mannose-binding protein A	32	6	5	25396
364	P41317	Mannose-binding protein C	25	6	5	25957
365	P28665	Murinoglobulin-1	38	50	26	165297

366	P28666	Murinoglobulin-2	14	20	1	162381
367	P04247	Myoglobin	21	2	2	17070
368	A0A2R8VK58	Myoglobin (Fragment)	39	2	2	9219
369	Q8VCS0	N-acetylmuramoyl-L-alanine amidase	12	5	5	57707
370	A0A571BF46	Nebulin	0	2	2	867113
371	A0A571BF58	Nebulin	0	2	2	871252
372	P32848	Parvalbumin alpha	33	3	3	11931
373	G5E898	Periplakin	0	1	1	203778
374	Q61171	Peroxiredoxin-2	57	10	10	21779
375	P16301	Phosphatidylcholine-sterol acyltransferase	16	5	5	49747
376	Q8VCU2	Phosphatidylinositol-glycan-specific phospholipase D	31	21	21	93624
377	A2A5K2	Phospholipid transfer protein	7	2	2	49023
378	P55065	Phospholipid transfer protein	6	2	2	54453
379	P97298	Pigment epithelium-derived factor	30	10	10	46234
380	P26262	Plasma kallikrein	41	22	22	71383
381	P97290	Plasma protease C1 inhibitor	28	12	12	55585
382	P20918	Plasminogen	56	38	37	90808
383	Q9Z126	Platelet factor 4	40	5	5	11243
384	O35930	Platelet glycoprotein Ib alpha chain	7	4	4	80055
385	Q60963	Platelet-activating factor acetylhydrolase	8	3	3	49258
386	Q9QXS1	Plectin	1	4	4	534193
387	A0A0G2JET4	Predicted gene 42543 (Fragment)	13	1	1	13114
388	A0A0N4SVU1	Predicted gene 7298	12	17	1	165287
389	A0A075B5J6	Predicted gene, 20730	11	1	1	13072
390	Q61838	Pregnancy zone protein	66	77	73	165852
391	P11680	Properdin	26	9	8	50327
392	Q9Z1R9	Protease, serine 1 (trypsin 1)	8	1	1	26135
393	Q07456	Protein AMBP	23	8	7	39029
394	Q8R121	Protein Z-dependent protease inhibitor	23	7	6	51797
395	P19221	Prothrombin	43	28	27	70269
396	Q2PZL6	Protocadherin Fat 4	0	1	1	540322

397	Q8CI01	Proz protein	11	2	2	26837
398	P52480	Pyruvate kinase PKM	10	5	5	57845
399	Q00724	Retinol-binding protein 4	54	9	9	23206
400	H7BWY6	Retinol-binding protein 4	44	9	9	28405
401	B2RX47	RIKEN cDNA 4930433I11 gene	2	1	1	69804
402	A0A0U1RPT6	RIKEN cDNA 4930433I11 gene	2	1	1	79455
403	Q8CJ40	Rootletin	1	3	2	226942
404	P70274	Selenoprotein P	22	8	7	42706
405	P07759	Serine protease inhibitor A3K	54	23	6	46880
406	A0A0R4J0I1	Serine protease inhibitor A3K	39	15	1	46673
407	Q03734	Serine protease inhibitor A3M	44	18	7	47064
408	Q91WP6	Serine protease inhibitor A3N	42	15	9	46718
409	Q921I1	Serotransferrin	75	57	53	76724
410	P31532	Serum amyloid A-4 protein	34	4	4	15088
411	P12246	Serum amyloid P-component	39	8	8	26247
412	P52430	Serum paraoxonase/arylesterase 1	59	17	17	39565
413	Q8BND5	Sulfhydryl oxidase 1	14	10	10	82785
414	P08228	Superoxide dismutase [Cu-Zn]	23	3	3	15943
415	P43025	Tetranectin	7	1	1	22257
416	P10639	Thioredoxin	9	1	1	11675
417	P35441	Thrombospondin-1	20	19	19	129647
418	O88968	Transcobalamin-2	3	1	1	47586
419	P07309	Transthyretin	62	6	6	15776
420	E9QNP0	Ubiquitin-60S ribosomal protein L40	22	4	4	26856
421	E9Q6R7	Utrophin	0	2	2	392707
422	Q9QZ25	Vascular non-inflammatory molecule 3	12	5	5	56305
423	P21614	Vitamin D-binding protein	55	23	22	53600
424	Q9CQW3	Vitamin K-dependent protein Z	7	2	2	44304
425	P29788	Vitronectin	31	12	12	54849
426	Q4U4S6	Xin actin-binding repeat-containing protein 2	0	3	2	428262
427	A0A5F8MPR4	Xin actin-binding repeat-containing protein 2	0	3	2	397309

428	E9QMD3	Zinc finger homeobox protein 3	1	3	3	406109
429	Q64726	Zinc-alpha-2-glycoprotein	39	13	13	35332

Table S3. Description of the top 10 most abundant proteins present in the CN, NT20, and T20 subgroups during experimental infection in the acute and chronic phases with the different *T. cruzi* strains Be-78 and VL-10.

N°	%	CN Acute	%	Be-78 NT20 Acute	%	Be-78 T20 Acute
1	74,62	Albumin	69,12	Albumin	73,18	Albumin
2	4,84	Serotransferrin	5,81	Serotransferrin	5,25	Serotransferrin
3	3,70	Protein da zona gestational	4,52	Protein da zona gestational	4,87	Protein da zona gestational
4	2,12	Apolipoprotein A-I	2,08	Hemopexin	2,49	Apolipoprotein A-I
5	1,67	Complement C3	1,92	Apolipoprotein A-I	1,48	Complement C3
6	1,46	Alpha globin 1	1,61	Immunoglobulin kappa constant	1,46	Hemopexin
7	1,02	Hemopexin	1,26	Alpha globin 1	1,37	Alpha globin 1
8	0,74	Serine protease inhibitor A3K	1,22	Complement C3	0,80	Serine protease inhibitor A3K
9	0,73	Beta-globin	1,13	Immunoglobulin heavy constant gamma	0,72	Alpha-1B-glycoprotein
10	0,62	Fibrinogen beta chain	0,87	Immunoglobulin heavy constant um	0,52	Beta-globin
**N°**	**%**	**CN Acute**	**%**	**VL-10 NT20 Acute**	**%**	**VL-10 T20 Acute**
1	74,70	Albumin	64,29	Albumin	71,87	Albumin
2	4,77	Serotransferrin	6,20	Serotransferrin	5,37	Serotransferrin
3	3,67	Pregnancy zone protein	6,08	Pregnancy zone protein	4,45	Pregnancy zone protein
4	2,19	Apolipoprotein A-I	2,54	Hemopexin	2,38	Apolipoprotein A-I
5	1,68	Complement C3	1,94	Apolipoprotein A-I	1,33	Hemopexin
6	1,45	Alpha globin 1	1,90	Immunoglobulin heavy constant gamma	1,32	Alpha globin 1
7	1,01	Hemopexin	1,87	Immunoglobulin kappa constant	1,27	Immunoglobulin heavy constant
8	0,74	Serine protease inhibitor A3K	1,84	Complement C3	1,20	Complement C3
9	0,72	Beta-globin	1,11	Alpha globin 1	1,15	Immunoglobulin kappa constant
10	0,68	Fibrinogen beta chain	1,03	Immunoglobulin heavy constant um	0,70	Serine protease inhibitor A3K

N°	%	CN Chronic	%	Be-78 NT20 Chronic	%	Be-78 T20 Chronic
1	73,05	Albumin	69,07	Albumin	73,70	Albumin
2	4,43	Pregnancy zone protein	5,16	Serotransferrin	4,81	Pregnancy zone protein
3	4,12	Serotransferrin	4,88	Pregnancy zone protein	3,58	Serotransferrin
4	2,46	Alpha globin 1	2,07	Immunoglobulin heavy constant gamma	2,87	Immunoglobulin kappa constant
5	2,35	Apolipoprotein A-I	1,91	Apolipoprotein A-I	1,80	Apolipoprotein A-I
6	1,67	Immunoglobulin kappa consta	1,38	Hemopexin	1,06	Immunoglobulin heavy constant
7	0,88	Complement C3	1,31	Immunoglobulin kappa constant	1,00	Complement C3
8	0,85	Hemopexin	1,02	Alpha globin 1	0,88	Hemopexin
9	0,78	Alpha-2-HS-glycoprotein	0,80	Complement C3	0,70	Alpha-1B-glycoprotein
10	0,50	Alpha-1B-glycoprotein	0,75	Alpha-1B-glycoprotein	0,70	Alpha-2-HS-glycoprotein
**N°**	**%**	**CN Chronic**	**%**	**VL-10 NT20 Chronic**	**%**	**VL-10 T20 Chronic**
1	75,11	Albumin	64,96	Albumin	70,60	Albumin
2	4,48	Pregnancy zone protein	6,39	Pregnancy zone protein	5,34	Pregnancy zone protein
3	4,21	Serotransferrin	4,79	Serotransferrin	4,68	Serotransferrin
4	2,41	Alpha globin 1	3,62	Immunoglobulin heavy constant gamma	2,11	Immunoglobulin heavy constant
5	1,72	Apolipoprotein A-I	2,27	Immunoglobulin kappa constant	1,97	Apolipoprotein A-I
6	0,90	Immunoglobulin kappa	2,14	Immunoglobulin heavy constant um	1,62	Hemopexin
7	0,87	Complement C3	1,56	Apolipoprotein A-I	1,43	Immunoglobulin kappa constant
8	0,80	Hemopexin	1,44	Hemopexin	1,08	Immunoglobulin heavy constant um
9	0,51	Alpha-2-HS-glycoprotein	1,08	Complement C3	0,99	Complement C3
10	0,42	Alpha-1B-glycoprotein	0,94	Alpha-1B-glycoprotein	0,45	Alpha-1B-glycoprotein

Table S4. Description of proteins found exclusively in the CN, NT20, and T20 subgroups of mice experimentally infected with the Be-78 and VL-10 *T. cruzi* strains during the acute and chronic phases.

N°	CN Be-78 Acute	CN Be-78 Chronic	CN VL-10 Acute	CN VL-10 Chronic
1	Beta-enolase	Creatine kinase M-type	Bisphosphoglycerate mutase	Apolipoprotein C-II
2	Coagulation factor XIII A chain	Heat shock cognate 71 kDa protein	Coagulation factor XIII A chain	Clusterin (Fragment)
3	Complement C5	Ig heavy chain V region	Fatty acid-binding protein liver	Coagulation factor V
4	Elongation factor 1-alpha 1	Immunoglobulin heavy variable 2-2 (Fragment)	Fibrous sheath-interacting protein 2	Creatine kinase M-type
5	FAT atypical cadherin 1	Immunoglobulin kappa chain variable 4-61	Hemoglobin subunit beta-H0	E3 ubiquitin-protein ligase RNF213
6	Fatty acid-binding protein liver	Immunoglobulin kappa chain variable 6-25 (Fragment	Hemoglobin subunit beta-H1	Immunoglobulin heavy variable 2-2 (Fragment)
7	Fibrinogen beta chain	Immunoglobulin kappa variable 6-14 (Fragment)	Histone H3-lysine (4) N-methyltransferase	Immunoglobulin kappa chain variable 4-61
8	Fibrinogen gamma chain	Immunoglobulin kappa variable 6-23 (Fragment)	Histone-lysine N-methyltransferase 2C	Immunoglobulin kappa chain variable 6-25 (Fragment)
9	Heat shock cognate 71 kDa protein	Myoglobin	Keratin type I cytoskeletal 16	Immunoglobulin kappa variable 6-14 (Fragment)
10	Hemoglobin subunit beta-H0	Myoglobin (Fragment)	Keratin, type I cytoskeletal 13	Immunoglobulin kappa variable 6-23 (Fragment)
11	Hemoglobin subunit beta-H1	Triosephosphate isomerase	Keratin, type I cytoskeletal 15	Transformation/transcription domain-associated protein
12	Keratin type I cytoskeletal 16		Periostin	

13	Keratin, type I cytoskeletal 13		Phosphoglycerate kinase 1	
14	Keratin, type II cuticular Hb4		Platelet glycoprotein V	
15	Keratin, type II cytoskeletal 8		Platelet glycoprotein V (Fragment)	
16	Periostin		Tubulin beta-2A chain	
17	Phosphoglycerate kinase 1		Tubulin beta-2B chain	
18	Phosphoglycerate mutase 2		Tubulin beta-4A chain	
19	Predicted gene 7298		Tubulin beta-4B chain	
20	Tubulin beta-2A chain		Tubulin beta-5 chain	
21	Tubulin beta-2B chain			
22	Tubulin beta-4B chain			
23	Tubulin beta-5 chain			

**N°**	**NT20 Be-78 Acute**	**NT20 Be-78 Chronic**	**NT20 VL-10 Acute**	**NT20 VL-10 Chronic**
1	C-C motif chemokine 8	Complement C2	Cathepsin S	Alpha-hemoglobin-stabilizing protein
2	Immunoglobulin heavy variable 2-6 (Fragment)	Complement factor H-related 4	C-C motif chemokine 8	Apolipoprotein C-IV
3	Cathepsin S	C-type lectin domain family 3, member b	Clusterin (Fragment)	Chymotrypsin-like elastase family member 2ª
4	Chymotrypsin-like elastase family member 2ª	EGF-containing fibulin-like extracellular matrix protein 1	Complement component 6	Glutathione S-transferase Mu 1
5	Clusterin (Fragment)	Histocompatibility 2, Q region locus 4	Complement component C6	Glutathione S-transferase Mu 1 (Fragment)
6	Complement C2	Ig gamma-2A chain C region A allele	Ezrin	Glutathione transferase

7	Cytosol aminopeptidase	Ig gamma-2A chain C region, membrane-bound form	Ig heavy chain V region 1-62-3	Histocompatibility 2, Q region locus 4
8	Ig gamma-1 chain C region secreted form	Ig heavy chain V region J539	Ig heavy chain V region 1-72	Ig heavy chain V region 23
9	Ig gamma-1 chain C region, membrane-bound form	Ig kappa chain V-I region S107A	Ig heavy chain V region 23	Ig heavy chain V region 36-65
10	Ig heavy chain V region	Ig kappa chain V-V region HP 124E1	Ig heavy chain V region 3	Ig heavy chain V region 914
11	Ig heavy chain V region 102	Immunoglobulin heavy variable 10-1 (Fragment)	Ig heavy chain V-III region E109	Ig heavy chain V region 93G7
12	Ig heavy chain V region 1-62-3	Immunoglobulin heavy variable 11-1 (Fragment)	Ig kappa chain V-I region S107A	Ig heavy chain V region J539
13	Ig heavy chain V region 1-72	Immunoglobulin heavy variable 1-58 (Fragment)	Ig kappa chain V-III region M	Ig kappa chain V-II region 26-10
14	Ig heavy chain V region 3	Immunoglobulin heavy variable 1-82	Ig kappa chain V-III region PC 2880/PC 1229	Ig kappa chain V-III region PC 3741/TEPC 111
15	Ig heavy chain V region 36-65	Immunoglobulin heavy variable 1-82 (Fragment)	Ig lambda-1 chain V region	Ig kappa chain V-III region PC 6308
16	Ig heavy chain V region 5-76	Immunoglobulin heavy variable 5-4 (Fragment)	Ig lambda-1 chain V region S43	Ig kappa chain V-V regions
17	Ig heavy chain V region 914	Immunoglobulin heavy variable 7-4	Immunoglobulin heavy variable 1-36	Immunoglobulin heavy variable 5-15
18	Ig heavy chain V region 93G7	Immunoglobulin heavy variable 7-4 (Fragment)	Immunoglobulin heavy variable 1-36 (Fragment)	Immunoglobulin heavy variable 9-1
19	Ig heavy chain V-III region E109	Immunoglobulin heavy variable V10-3 (Fragment)	Immunoglobulin heavy variable 1-52	Immunoglobulin heavy variable V1-42

20	Ig kappa chain V region Mem5 (Fragment)	Immunoglobulin heavy variable V11-2 (Fragment)	Immunoglobulin heavy variable 1-52 (Fragment)	Immunoglobulin kappa chain variable 4-54
21	Ig kappa chain V-I region S107A	Immunoglobulin heavy variable V1-20	Immunoglobulin heavy variable 1-53 (Fragment)	Immunoglobulin kappa chain variable 5-43 (Fragment)
22	Ig kappa chain V-I region S107A (Fragment)	Immunoglobulin heavy variable V1-20 (Fragment)	Immunoglobulin heavy variable 1-64 (Fragment)	Immunoglobulin kappa variable 1-135 (Fragment)
23	Ig kappa chain V-II region M	Immunoglobulin heavy variable V1-43	Immunoglobulin heavy variable 1-75 (Fragment)	Insulin-like growth factor-binding protein 3
24	Ig kappa chain V-III region M	Immunoglobulin heavy variable V1-7 (Fragment)	Immunoglobulin heavy variable 2-6 (Fragment)	Nebulin
25	Ig kappa chain V-III region PC 2880/PC 1229	Immunoglobulin kappa chain variable 12-41 (Fragment)	Immunoglobulin heavy variable 3-1 (Fragment)	Proteasome activator complex subunit 4
26	Ig kappa chain V-III region PC 3741/TEPC 111	Immunoglobulin kappa variable 10-94 (Fragment)	Immunoglobulin heavy variable 9-4	
27	Ig kappa chain V-III region PC 6684	Immunoglobulin kappa variable 8-18 (Fragment)	Immunoglobulin heavy variable V1-11 (Fragment)	
28	Ig kappa chain V-III region PC 7175	Immunoglobulin kappa variable 8-19	Immunoglobulin heavy variable V1-12	
29	Ig kappa chain V-V region HP 124E1	Immunoglobulin kappa variable 8-26 (Fragment)	Immunoglobulin heavy variable V1-12 (Fragment)	
30	Ig kappa chain V-VI region NQ2-17,4,1	Keratin type II cytoskeletal 5	Immunoglobulin heavy variable V15-2	
31	Ig lambda-1 chain V region	Keratin, type I cytoskeletal 42	Immunoglobulin heavy variable V15-2 (Fragment)	
32	Ig lambda-1 chain V region S43	Tetranectin	Immunoglobulin heavy variable V1-59	

33	Immunoglobulin heavy variable 13-2	Immunoglobulin heavy variable V1-59 (Fragment)
34	Immunoglobulin heavy variable 13-2 (Fragment)	Immunoglobulin heavy variable V1-63 (Fragment)
35	Immunoglobulin heavy variable 1-36	Immunoglobulin kappa chain variable 1-88 (Fragment)
36	Immunoglobulin heavy variable 1-36 (Fragment)	Immunoglobulin kappa chain variable 6-17
37	Immunoglobulin heavy variable 14-1 (Fragment)	Immunoglobulin kappa chain variable 8-24 (Fragment)
38	Immunoglobulin heavy variable 14-4	Immunoglobulin kappa chain variable 8-30 (Fragment)
39	Immunoglobulin heavy variable 14-4 (Fragment)	Immunoglobulin kappa variable 10-94 (Fragment)
40	Immunoglobulin heavy variable 1-50 (Fragment)	Immunoglobulin kappa variable 4-81
41	Immunoglobulin heavy variable 1-52	Immunoglobulin kappa variable 8-21 (Fragment)
42	Immunoglobulin heavy variable 1-52 (Fragment)	Immunoglobulin lambda variable 1 (Fragment)
43	Immunoglobulin heavy variable 1-53 (Fragment)	Insulin-like growth factor-binding protein 3
44	Immunoglobulin heavy variable 1-58 (Fragment)	Junction plakoglobin
45	Immunoglobulin heavy variable 1-64 (Fragment)	Keratin, type II cytoskeletal 73

46	Immunoglobulin heavy variable 1-66 (Fragment)	Microtubule-actin cross-linking factor 1, isoforms 1/2/3/4
47	Immunoglobulin heavy variable 1-75 (Fragment)	Periplakin
48	Immunoglobulin heavy variable 1-77	Plasma membrane calcium-transporting ATPase 2
49	Immunoglobulin heavy variable 1-77 (Fragment)	Predicted gene 42543 (Fragment)
50	Immunoglobulin heavy variable 1-80 (Fragment)	Proteasome subunit alpha type-6
51	Immunoglobulin heavy variable 1-85 (Fragment)	Protein SFI1 homolog
52	Immunoglobulin heavy variable 3-1 (Fragment)	Serpin A11
53	Immunoglobulin heavy variable 5-15	Vascular cell adhesion protein 1
54	Immunoglobulin heavy variable 5-15 (Fragment)	
55	Immunoglobulin heavy variable 5-6 (Fragment)	
56	Immunoglobulin heavy variable 5-9 (Fragment)	
57	Immunoglobulin heavy variable 9-1	
58	Immunoglobulin heavy variable 9-4	

59 Immunoglobulin heavy variable V1-12

60 Immunoglobulin heavy variable V1-12 (Fragment)

61 Immunoglobulin heavy variable V1-19

62 Immunoglobulin heavy variable V1-19 (Fragment)

63 Immunoglobulin heavy variable V1-42

64 Immunoglobulin heavy variable V1-43

65 Immunoglobulin heavy variable V14-3 (Fragment)

66 Immunoglobulin heavy variable V1-43 (Fragment)

67 Immunoglobulin heavy variable V15-2

68 Immunoglobulin heavy variable V15-2 (Fragment)

69 Immunoglobulin heavy variable V1-59

70 Immunoglobulin heavy variable V1-59 (Fragment)

71 Immunoglobulin heavy variable V1-67

72 Immunoglobulin heavy variable V1-67 (Fragment)

73 Immunoglobulin heavy variable V1-74

74 Immunoglobulin heavy variable V1-74 (Fragment)

75 Immunoglobulin heavy variable V9-3 (Fragment)

76 Immunoglobulin kappa chain variable 12-41 (Fragment)

77 Immunoglobulin kappa chain variable 13-84 (Fragment)

78 Immunoglobulin kappa chain variable 13-85 (Fragment)

79 Immunoglobulin kappa chain variable 4-51 (Fragment)

80 Immunoglobulin kappa chain variable 6-25 (Fragment)

81 Immunoglobulin kappa chain variable 8-24 (Fragment)

82 Immunoglobulin kappa chain variable 8-30 (Fragment)

83 Immunoglobulin kappa variable 10-94 (Fragment)

84 Immunoglobulin kappa variable 14-130

85	Immunoglobulin kappa variable 14-130 (Fragment)
86	Immunoglobulin kappa variable 2-112
87	Immunoglobulin kappa variable 3-1 (Fragment)
88	Immunoglobulin kappa variable 4-57-1 (Fragment)
89	Immunoglobulin kappa variable 4-80 (Fragment)
90	Immunoglobulin kappa variable 5-37 (Fragment)
91	Immunoglobulin kappa variable 6-13
92	Immunoglobulin kappa variable 6-23 (Fragment)
93	Immunoglobulin kappa variable 8-21 (Fragment)
94	Immunoglobulin kappa variable 8-28
95	Immunoglobulin kappa variable 8-28 (Fragment)
96	Immunoglobulin kappa variable 9-123 (Fragment)
97	Immunoglobulin lambda variable 1 (Fragment)

98	Immunoglobulin lambda variable 3 (Fragment)
99	Insulin-like growth factor-binding protein 3
100	Keratin-like protein KRT222
101	Lipopolysaccharide-binding protein
102	Proteasome subunit alpha type-2
103	Proteasome subunit alpha type-2 (Fragment)
104	Purine nucleoside phosphorylase
105	Vascular cell adhesion protein 1

N°	T20 Be-78 Acute	T20 Be-78 Chronic	T20 VL-10 Acute	T20 VL-10 Chronic
1	Centromere protein F	Carboxylesterase 1D	Keratin, type I cytoskeletal 17	Alpha-1-antitrypsin 1-5
2	Centrosome-associated protein CEP250	Immunoglobulin kappa chain variable 20-101-2 (Fragment)	Immunoglobulin heavy variable V9-2 (Fragment)	Apolipoprotein N
3	Keratin, type I cytoskeletal 14	Carboxylesterase 1E	Nebulin	Complement factor H-related 1
4	Centrosomal protein of 162 kDa	Cathepsin B	Ig heavy chain V region AC38 205,12	Complement factor H-related 2
5	Cathepsin B	Serine/threonine-specific protein kinase		Fibrinogen alpha chain
6	Junction plakoglobin			Ig heavy chain V region AC38 205,12
7	von Willebrand factor			Ig heavy chain V region TEPC 1017

8	Ig kappa chain V-I region S107A
9	Ig kappa chain V-I region S107A (Fragment)
10	Ig kappa chain V-V region M
11	Immunoglobulin heavy variable 11-1 (Fragment)
12	Immunoglobulin heavy variable 1-52
13	Immunoglobulin heavy variable 1-52 (Fragment)
14	Immunoglobulin heavy variable 1-58 (Fragment)
15	Immunoglobulin heavy variable 1-75 (Fragment)
16	Immunoglobulin heavy variable 1-78
17	Immunoglobulin heavy variable 1-78 (Fragment)
18	Immunoglobulin heavy variable 7-2
19	Immunoglobulin heavy variable 7-2 (Fragment)
20	Immunoglobulin heavy variable 7-4
21	Immunoglobulin heavy variable 7-4 (Fragment)

22	Immunoglobulin heavy variable V11-2 (Fragment)
23	Immunoglobulin kappa chain variable 9-120
24	Immunoglobulin kappa chain variable 9-120 (Fragment)
25	Immunoglobulin kappa variable 10-94 (Fragment)
26	Immunoglobulin kappa variable 4-53
27	Immunoglobulin kappa variable 4-53 (Fragment)
28	Keratin, type II cytoskeletal 5
29	Predicted gene 7298
30	Rootletin

Table S5. Differentially abundant proteins in the Be-78 strain during the acute phase

N°	Access	Log2 Ratio			Description
		CN	NT20	T20	
1	B7ZCG3	21,7	10,6	1,0	BPI fold-containing family A member 2 (Fragment)
2	F6WR04	0,1	1,0	0,0	Cathepsin S
3	Q9QWK4	0,3	2,3	1,0	CD5 antigen-like
4	P98086	0,6	2,6	1,0	Complement C1q subcomponent subunit A
5	P14106	0,7	2,6	1,0	Complement C1q subcomponent subunit B
6	Q06770	0,4	0,7	1,0	Corticosteroid-binding globulin
7	Q8HWB2	0,1	4,3	1,0	Histocompatibility 2 Q region locus 4
8	P18531	0,5	4,2	1,0	Immunoglobulin heavy chain V region 3-6
9	A0A075B6A3	0,7	2,3	1,0	Immunoglobulin heavy constant alpha (Fragment)
10	A0A075B5R6	0,0	4,6	1,0	Immunoglobulin heavy variable 11-1 (Fragment)
11	A0A087WPN7	0,2	3,3	1,0	Immunoglobulin heavy variable 13-2
12	A0A075B5V5	0,0	39,2	1,0	Immunoglobulin heavy variable 1-39 (Fragment)
13	A0A075B5R7	0,6	32,9	1,0	Immunoglobulin heavy variable 14-2 (Fragment)
14	A0A075B5V8	0,4	3,7	1,0	Immunoglobulin heavy variable 1-47
15	A0A075B680	0,0	5,6	1,0	Immunoglobulin heavy variable 1-62-2
16	A0A0B4J1N0	0,0	6,2	1,0	Immunoglobulin heavy variable 1-76
17	A0A075B5Y3	0,0	19,2	1,0	Immunoglobulin heavy variable 1-80 (Fragment)
18	A0A075B5P8	0,1	13,8	1,0	Immunoglobulin heavy variable 2-2 (Fragment)
19	A0A075B5Q3	0,0	23,9	1,0	Immunoglobulin heavy variable 2-5
20	A0A075B6A7	0,1	1,0	0,0	Immunoglobulin heavy variable 2-6 (Fragment)
21	A0A075B697	0,0	57,5	1,0	Immunoglobulin heavy variable 2-9-1 (Fragment)
22	A0A075B5R5	0,2	1,9	1,0	Immunoglobulin heavy variable 4-1 (Fragment)
23	A0A075B5R0	0,2	7,4	1,0	Immunoglobulin heavy variable 5-16
24	A0A0A6YVS4	0,2	7,3	1,0	Immunoglobulin heavy variable 5-9-1 (Fragment)
25	A0A075B5S2	0,5	2,7	1,0	Immunoglobulin heavy variable 7-1 (Fragment)
26	A0A075B5R2	0,1	4,4	1,0	Immunoglobulin heavy variable 7-3 (Fragment)
27	A0A0A6YXQ0	0,0	37,9	1,0	Immunoglobulin heavy variable 8-8 (Fragment)
28	A0A075B5T6	0,0	29,1	1,0	Immunoglobulin heavy variable V10-3 (Fragment)
29	A0A0A6YWI9	0,0	0,2	1,0	Immunoglobulin heavy variable V1-11 (Fragment)
30	A0A075B5U6	0,0	15,9	1,0	Immunoglobulin heavy variable V1-20
31	A0A075B5V6	0,0	18,5	1,0	Immunoglobulin heavy variable V1-42
32	A0A075B5T5	0,4	15,5	1,0	Immunoglobulin heavy variable V1-5
33	A0A075B5Y1	0,0	19,3	1,0	Immunoglobulin heavy variable V1-74
34	A0A075B5T9	0,0	7,6	1,0	Immunoglobulin heavy variable V1-9
35	A0A0G2JDE1	0,0	12,4	1,0	Immunoglobulin heavy variable V8-12 (Fragment)
36	P01592	0,1	6,9	1,0	Immunoglobulin J chain
37	A0A140T8P3	0,4	5,5	1,0	Immunoglobulin kappa chain variable 15-103
38	A0N8I8	0,0	16,2	1,0	Immunoglobulin kappa chain variable 4-51
39	A0A0B4J1J1	0,1	12,9	1,0	Immunoglobulin kappa chain variable 5-45

40	A0A075B5K2	0,3	8,6	1,0	Immunoglobulin kappa chain variable 9-124
41	P01668	0,3	18,0	1,0	Immunoglobulin kappa chain V-III region PC
42	P01678	0,1	5,3	1,0	Immunoglobulin kappa chain V-VI region SAPC
43	A0A140T8M0	0,0	4,9	1,0	Immunoglobulin kappa variable 1-117 (Fragment)
44	A0A140T8M8	0,0	2,5	1,0	Immunoglobulin kappa variable 1-131 (Fragment)
45	A0A0B4J1H7	0,1	4,4	1,0	Immunoglobulin kappa variable 1-135 (Fragment)
46	A0A140T8M2	0,3	7,3	1,0	Immunoglobulin kappa variable 12-44 (Fragment)
47	A0A140T8P6	0,3	7,2	1,0	Immunoglobulin kappa variable 12-46 (Fragment)
48	A0A075B5K0	0,0	4,9	1,0	Immunoglobulin kappa variable 14-126 (Fragment)
49	A0A0B4J1I1	0,0	13,1	1,0	Immunoglobulin kappa variable 16-104 (Fragment)
50	A0A075B5K3	0,2	8,1	1,0	Immunoglobulin kappa variable 17-121
51	A0A075B677	0,2	8,7	1,0	Immunoglobulin kappa variable 4-53
52	A0A075B5M4	0,2	8,8	1,0	Immunoglobulin kappa variable 4-57-1 (Fragment)
53	A0A0B4J1K5	0,1	1,0	0,0	Immunoglobulin lambda variable 3 (Fragment)
54	P01844	0,1	2,2	1,0	Immunoglobulin lambda-2 chain C region
55	A0A0G2JET4	0,0	19,0	1,0	Predicted gene 42543 (Fragment) (Ig)
56	A0A0G2JGT0	0,0	0,1	1,0	Predicted gene 43218 (Fragment) (Ig)
57	A6XA75	0,2	0,1	1,0	Trem-like transcript 1 protein (Ig)
58	P29533	1,0	9,2	0,0	Vascular cell adhesion protein 1
59	P17182	1,0	0,9	0,0	Alpha-enolase
60	P34928	0,4	0,6	1,0	Apolipoprotein C-I
61	Q61268	0,7	0,3	1,0	Apolipoprotein C-IV
62	Q8BH61	21,2	0,0	1,0	Coagulation factor XIII A chain
63	B7ZNJ1	14,7	3,4	1,0	Fibronectin
64	P06151	1,0	0,2	0,0	L-lactate dehydrogenase A chain
65	P55065	0,1	0,4	1,0	Phospholipid transfer protein
66	Q9Z126	0,3	0,5	1,0	Platelet factor 4
67	E9PVU0	0,2	0,0	1,0	Unconventional myosin-VI

Table S6. Differentially abundant proteins in the Be-78 strain during the chronic phase

N°	Access	Log2 Ratio			Description
		CN	NT20	T20	
1	P98086	1,0	33,6	44,9	Complement C1q subcomponent subunit A
2	P01844	1,0	3,7	1,8	Immunoglobulin lambda-2 chain C region
3	A0A075B5R6	1,0	7,1	0,2	Immunoglobulin heavy variable 11-1 (Fragment)
4	A0A087WPN7	0,0	0,6	1,0	Immunoglobulin heavy variable 13-2
5	A0A075B5V8	1,0	8,0	2,7	Immunoglobulin heavy variable 1-47
6	A0A075B5W6	1,0	0,3	0,0	Immunoglobulin heavy variable 1-55 (Fragment)
7	A0A075B680	1,0	0,0	0,0	Immunoglobulin heavy variable 1-62-2
8	A0A140T8P5	1,0	5,3	4,0	Immunoglobulin kappa chain variable 8-24 (Fragment)
9	A0A0G2JEY5	1,0	6,2	0,2	Immunoglobulin kappa variable 4-81 (Fragment)
10	A0A0R4J268	0,0	2,7	1,0	Oncostatin-M-specific receptor subunit beta
11	P13634	1,0	0,5	0,0	Carbonic anhydrase 1
12	P07310	1,0	0,1	0,0	Creatine kinase M-type
13	A0A3B2W4E4	1,0	15,3	20,9	EMILIN-2
14	Q9JI57	1,0	0,3	0,0	General transcription factor II-I repeat domain
15	Q3UWA6	0,0	0,7	1,0	Guanylyl cyclase C
16	Q3V1J8	1,0	0,1	0,8	Hyaluronan-binding protein 2
17	A0A0G2JGQ4	1,0	0,0	12,8	NEDD8 ultimate buster 1
18	P32848	1,0	0,5	0,1	Parvalbumin alpha
19	E9PVX6	1,0	11,3	8,4	Proliferation marker protein Ki-67
20	Q3TDN0	1,0	1,7	0,2	Protein dispatched homolog 1
21	F8WGW3	1,0	0,6	0,0	S1 RNA-binding domain-containing protein 1
22	Q3TMX7	1,0	7,2	6,9	Sulfhydryl oxidase 2

Table S7. Differentially abundant proteins in the VL-10 strain during the acute phase

N°	Access	Log2 Ratio			Description
		CN	NT20	T20	
1	F6WR04	0,0	1,0	0,2	Cathepsin S
2	Q9QWK4	1,0	16,8	2,8	CD5 antigen-like
3	P98086	1,0	5,9	3,6	Complement C1q subcomponent subunit A
4	P14106	1,0	6,0	3,3	Complement C1q subcomponent subunit B
5	E9Q6D8	1,0	112,6	12,8	Complement component C6
6	Q61646	1,0	28,8	0,2	Haptoglobin
7	Q8HWB2	1,0	75,8	19,2	Histocompatibility 2 Q region locus 4
8	A0A0B4J1J6	0,0	1,0	0,8	Immunoglobulin heavy variable 10-1
9	A0A075B5R6	0,0	1,0	0,8	Immunoglobulin heavy variable 11-1 (Fragment)
10	A0A087WPN7	0,0	1,0	0,5	Immunoglobulin heavy variable 13-2
11	A0A075B5R7	1,0	46,6	28,2	Immunoglobulin heavy variable 14-2 (Fragment)
12	A0A075B5S3	0,0	1,0	1,2	Immunoglobulin heavy variable 14-4
13	A0A075B680	0,0	1,0	0,3	Immunoglobulin heavy variable 1-62-2
14	A0A0B4J1M0	1,0	12,0	1,4	Immunoglobulin heavy variable 1-77
15	A0A075B5Y3	0,0	1,0	0,4	Immunoglobulin heavy variable 1-80 (Fragment)
16	A0A075B5Q3	0,0	1,0	0,1	Immunoglobulin heavy variable 2-5
17	A0A075B5R5	1,0	6,8	5,1	Immunoglobulin heavy variable 4-1 (Fragment)
18	A0A075B5R0	1,0	42,8	22,2	Immunoglobulin heavy variable 5-16
19	A0A0A6YVS4	1,0	39,1	15,8	Immunoglobulin heavy variable 5-9-1 (Fragment)
20	A0A075B5S2	1,0	13,2	2,4	Immunoglobulin heavy variable 7-1 (Fragment)
21	A0A075B5R2	1,0	16,2	4,3	Immunoglobulin heavy variable 7-3 (Fragment)
22	A0A0A6YXQ0	0,0	1,0	0,6	Immunoglobulin heavy variable 8-8 (Fragment)
23	A0A075B5U6	0,0	1,0	0,5	Immunoglobulin heavy variable V1-20
24	A0A075B5V6	0,0	1,0	1,3	Immunoglobulin heavy variable V1-42
25	A0A075B5T5	1,0	39,3	18,9	Immunoglobulin heavy variable V1-5
26	A0A075B5Y1	0,0	1,0	0,6	Immunoglobulin heavy variable V1-74
27	A0A075B5T9	1,0	106,2	53,4	Immunoglobulin heavy variable V1-9
28	A0A0G2JDE1	0,0	1,0	0,4	Immunoglobulin heavy variable V8-12 (Fragment)
29	P01592	1,0	80,8	7,2	Immunoglobulin J chain
30	P01678	1,0	145,0	151,0	Immunoglobulin kappa chain V-VI region SAPC 10
31	A0A0B4J1H7	1,0	64,1	57,5	Immunoglobulin kappa variable 1-135 (Fragment)
32	A0A140T8M2	1,0	25,0	8,0	Immunoglobulin kappa variable 12-44 (Fragment)
33	A0A140T8P6	1,0	37,8	14,6	Immunoglobulin kappa variable 12-46 (Fragment)
34	A0A075B5K0	1,0	67,6	43,8	Immunoglobulin kappa variable 14-126
35	A0A075B677	1,0	109,2	39,1	Immunoglobulin kappa variable 4-53
36	A0A075B664	0,0	1,0	0,7	Immunoglobulin lambda variable 2
37	P01844	1,0	33,5	28,9	Immunoglobulin lambda-2 chain C region

38	P11680	1,0	6,2	2,5	Properdin
39	P33622	1,0	0,8	0,2	Apolipoprotein C-III
40	Q8BH61	1,0	0,0	0,0	Coagulation factor XIII A chain
41	Q91X72	1,0	2,9	1,5	Hemopexin
42	P06151	1,0	16,5	9,2	L-lactate dehydrogenase A chain
43	E9PZF0	1,0	14,5	10,1	Nucleoside diphosphate kinase

Table S8. Differentially abundant proteins in the VL-10 strain during the chronic phase

N°	Access	Log2 Ratio			Description
		CN	NT20	T20	
1	B7ZCG3	1,0	5,1	6,6	BPI fold-containing family A member 2 (Fragment)
2	P08607	1,0	3,5	2,5	C4b-binding protein
3	P14106	1,0	10,3	8,2	Complement C1q subcomponent subunit B
4	P14847	1,0	9,3	20,9	C-reactive protein
5	A0A075B680	1,0	0,0	0,0	Immunoglobulin heavy variable 1-62-2
6	A0A0B4J1M0	0,0	1,0	0,2	Immunoglobulin heavy variable 1-77
7	A0A075B5R5	1,0	9,2	2,4	Immunoglobulin heavy variable 4-1 (Fragment)
8	A0A075B5Q6	1,0	13,4	12,1	Immunoglobulin heavy variable 5-9-1
9	A0A075B5T2	1,0	6,2	4,9	Immunoglobulin heavy variable 6-3 (Fragment)
10	A0A075B5T5	1,0	8,6	3,7	Immunoglobulin heavy variable V1-5
11	P01592	1,0	27,4	12,6	Immunoglobulin J chain
12	P01628	0,0	0,0	1,0	Immunoglobulin kappa chain V-II region M
13	P01668	1,0	11,9	1,4	Immunoglobulin kappa chain V-III region PC 7210
14	P01636	0,0	0,0	1,0	Immunoglobulin kappa chain V-V region M
15	P01678	1,0	105,0	6,0	Immunoglobulin kappa chain V-VI region SAPC 10
16	A0A140T8M0	1,0	10,8	11,8	Immunoglobulin kappa variable 1-117 (Fragment)
17	A0A140T8M8	1,0	10,4	12,3	Immunoglobulin kappa variable 1-131 (Fragment)
18	A0A075B5K0	1,0	0,0	0,8	Immunoglobulin kappa variable 14-126 (Fragment)
19	A0A075B5M1	1,0	4,7	1,7	Immunoglobulin kappa variable 4-63
20	A0A140T8M4	0,0	1,0	1,9	Immunoglobulin kappa variable 8-19
21	A0A140T8P7	0,0	1,0	0,0	Immunoglobulin kappa variable 8-21 (Fragment)
22	P01845	1,0	7,1	3,4	Immunoglobulin lambda-3 chain C region
23	A0A0R4J1T8	1,0	77,6	13,1	Beta-alanine-activating enzyme (Fragment)
24	D3Z5G7	1,0	1,0	5,4	Carboxylic ester hydrolase
25	E9QKK1	1,0	6,4	4,0	Centromere-associated protein E
26	P07310	1,0	0,1	0,1	Creatine kinase M-type
27	Q8BIE6	0,0	1,0	0,3	FERM domain-containing protein 4A
28	A2A513	1,0	5,0	11,7	Keratin type I cytoskeletal 10
29	P06151	1,0	149,1	63,9	L-lactate dehydrogenase A chain
30	P32848	1,0	1,1	0,0	Parvalbumin alpha
31	P08228	1,0	10,1	4,3	Superoxide dismutase [Cu-Zn]
32	K3W4L0	1,0	0,2	0,6	Unconventional myosin-XVIIIa

Table S9. Proteins shared between the Be-78 and VL-10 strains in the acute phase

Proteins	Access	Log2 Ratio					Description
		CN	Be-78 NT20	VL-10 NT20	Be-78 T20	VL-10 T20	
1	B7ZCG3	1,0	0,5	0,5	0,1	0,3	BPI fold-containing family A member 2 (Fragment)
2	F6WR04	0,0	1,0	1,2	0,0	0,2	Cathepsin S
3	Q9QWK4	1,0	7,5	9,7	3,1	1,7	CD5 antigen-like
4	P98086	1,0	4,3	5,9	1,7	3,6	Complement C1q subcomponent subunit A
5	P14106	1,0	3,9	6,1	1,5	3,3	Complement C1q subcomponent subunit B
6	E9Q6D8	1,0	116,0	151,1	10,0	10,1	Complement component C6
7	Q8HWB2	0,0	1,0	1,5	0,2	0,4	Histocompatibility 2 Q region locus 4
8	A0A087WPN7	0,0	1,0	1,1	0,0	0,5	Immunoglobulin heavy variable 13-2
9	A0A075B5V5	0,0	1,0	1,3	0,0	0,6	Immunoglobulin heavy variable 1-39 (Fragment)
10	A0A075B5R7	1,0	56,6	56,4	1,7	34,1	Immunoglobulin heavy variable 14-2 (Fragment)
11	A0A075B5V8	1,0	10,2	6,0	2,4	1,7	Immunoglobulin heavy variable 1-47
12	A0A075B680	0,0	1,0	1,1	0,2	0,3	Immunoglobulin heavy variable 1-62-2
13	A0A0B4J1N0	0,0	1,0	1,7	0,2	0,5	Immunoglobulin heavy variable 1-76
14	A0A0B4J1M0	1,0	8,2	12,0	1,9	1,4	Immunoglobulin heavy variable 1-77
15	A0A075B5Y3	0,0	1,0	1,6	0,1	0,4	Immunoglobulin heavy variable 1-80 (Fragment)
16	A0A075B697	0,0	1,0	0,9	0,0	0,4	Immunoglobulin heavy variable 2-9-1 (Fragment)
17	A0A075B5R5	1,0	8,3	6,8	4,4	5,2	Immunoglobulin heavy variable 4-1 (Fragment)
18	A0A075B5R0	1,0	32,1	42,8	4,3	22,2	Immunoglobulin heavy variable 5-16
19	A0A0A6YVS4	1,0	46,4	39,1	6,3	15,8	Immunoglobulin heavy variable 5-9-1 (Fragment)
20	A0A075B5S2	1,0	5,2	13,2	1,9	2,4	Immunoglobulin heavy variable 7-1 (Fragment)
21	A0A075B5R2	1,0	33,4	16,2	7,7	4,3	Immunoglobulin heavy variable 7-3 (Fragment)
22	A0A0A6YXQ0	0,0	1,0	1,3	0,0	0,8	Immunoglobulin heavy variable 8-8 (Fragment)
23	A0A075B5U6	0,0	1,0	0,7	0,1	0,3	Immunoglobulin heavy variable V1-20

24	A0A075B5V6	0,0	1,0	1,7	0,1	2,2	Immunoglobulin heavy variable V1-42
25	A0A075B5T5	1,0	44,1	39,3	2,8	18,9	Immunoglobulin heavy variable V1-5
26	A0A075B5Y1	0,0	1,0	1,5	0,1	0,9	Immunoglobulin heavy variable V1-74
27	A0A075B5T9	1,0	149,2	107,8	21,1	54,2	Immunoglobulin heavy variable V1-9
28	A0A0G2JDE1	0,0	1,0	1,3	0,1	0,6	Immunoglobulin heavy variable V8-12 (Fragment)
29	P01592	1,0	83,3	80,8	12,0	7,2	Immunoglobulin J chain
30	A0A0B4J1J1	1,0	143,3	150,0	10,9	58,4	Immunoglobulin kappa chain variable 5-45 (Fragment)
31	P01668	1,0	57,4	22,7	3,2	12,6	Immunoglobulin kappa chain V-III region PC 7210
32	P01678	1,0	72,0	149,0	12,7	146,0	Immunoglobulin kappa chain V-VI region SAPC 10
33	A0A0B4J1H7	1,0	60,4	64,1	13,9	57,5	Immunoglobulin kappa variable 1-135 (Fragment)
34	A0A140T8M2	1,0	22,8	25,0	3,2	8,0	Immunoglobulin kappa variable 12-44 (Fragment)
35	A0A140T8P6	1,0	27,6	37,5	3,8	14,5	Immunoglobulin kappa variable 12-46 (Fragment)
36	A0A075B5K0	1,0	140,5	65,4	31,6	40,1	Immunoglobulin kappa variable 14-126 (Fragment)
37	A0A075B5K3	1,0	36,1	15,1	4,5	14,7	Immunoglobulin kappa variable 17-121
38	A0A075B677	1,0	52,9	109,2	6,1	39,1	Immunoglobulin kappa variable 4-53
39	A0A075B664	0,0	1,0	3,9	1,6	2,7	Immunoglobulin lambda variable 2
40	A0A0B4J1K5	0,0	1,0	0,7	0,0	0,3	Immunoglobulin lambda variable 3 (Fragment)
41	P01844	1,0	23,7	32,0	10,6	27,4	Immunoglobulin lambda-2 chain C region
42	A0A0G2JET4	1,0	4,4	4,3	1,0	0,6	Predicted gene 42543 (Fragment)
43	A0A0G2JGT0	0,0	1,0	2,6	13,7	0,0	Predicted gene 43218 (Fragment)
44	P33622	1,0	0,4	0,8	0,6	0,2	Apolipoprotein C-III
45	Q8BH61	1,0	0,0	0,0	0,0	0,0	Coagulation factor XIII A chain
46	B7ZNJ1	1,0	0,2	1,3	0,1	0,9	Fibronectin
47	Q91X72	1,0	2,3	2,8	1,5	1,4	Hemopexin
48	P06151	1,0	0,5	16,5	0,1	9,2	L-lactate dehydrogenase A chain
49	P55065	1,0	3,3	4,9	8,4	5,9	Phospholipid transfer protein
50	Q5K2P8	1,0	0,1	0,2	0,0	0,2	Polyserase-2
51	Q923W1	1,0	0,4	0,0	2,8	1,5	Trimethylguanosine synthase

Table S10. *Proteins shared between the Be-78 and VL-10 strains in the chronic phase*

Proteins	Access	Log2 Ratio					Description
		CN	Be-78 NT20	VL-10 NT20	Be-78 T20	VL-10 T20	
1	P07361	0,0	1,0	0,0	0,2	0,1	Alpha-1-acid glycoprotein 2
2	Q3U108	0,0	1,0	4,2	0,0	9,4	AT-rich interactive domain-containing protein 5A
3	B7ZCG3	1,0	0,8	5,1	2,2	6,6	BPI fold-containing family A member 2 (Fragment)
4	P98086	1,0	33,6	129,0	44,9	82,8	Complement C1q subcomponent subunit A
5	P14106	1,0	4,4	10,4	1,6	8,2	Complement C1q subcomponent subunit B
6	Q02105	1,0	2,0	4,4	0,6	3,4	Complement C1q subcomponent subunit C
7	Q8CFG8	1,0	1,9	0,0	4,7	0,0	Complement C1s-1 subcomponent
8	P14847	1,0	2,3	7,1	4,5	15,5	C-reactive protein
9	E9Q555	1,0	0,4	0,1	0,4	0,0	E3 ubiquitin-protein ligase RNF213
10	P01798	1,0	0,1	1,7	1,0	1,9	Immunoglobulin heavy chain V-III region E109
11	P01630	1,0	3,7	4,0	0,6	5,1	Immunoglobulin kappa chain V-II region 7S34.1
12	P01628	0,0	0,0	0,0	1,0	3,5	Immunoglobulin kappa chain V-II region MPC 511
13	P01668	1,0	0,8	11,8	0,7	1,3	Immunoglobulin kappa chain V-III region PC 7210
14	P01636	0,0	0,0	0,0	0,0	1,0	Immunoglobulin kappa chain V-V region MPC 149
15	P01678	1,0	2,6	105,0	7,9	7,4	Immunoglobulin kappa chain V-VI region SAPC 10
16	P01844	1,0	3,7	6,2	1,8	3,3	Immunoglobulin lambda-2 chain C region
17	A0A075B5R6	1,0	7,1	1,0	0,2	2,6	Immunoglobulin heavy variable 11-1 (Fragment)
18	A0A075B5V8	1,0	34,4	36,5	4,7	15,1	Immunoglobulin heavy variable 1-47
19	A0A075B5W6	1,0	0,3	0,5	0,0	0,3	Immunoglobulin heavy variable 1-55 (Fragment)
20	A0A075B680	1,0	0,0	0,0	0,0	0,0	Immunoglobulin heavy variable 1-62-2
21	A0A0B4J1M0	0,0	1,0	18,1	0,0	3,6	Immunoglobulin heavy variable 1-77
22	A0A075B5Y6	1,0	1,2	10,8	6,6	19,2	Immunoglobulin heavy variable 1-85 (Fragment)
23	A0A075B5R5	1,0	3,0	11,9	1,5	3,2	Immunoglobulin heavy variable 4-1 (Fragment)

24	A0A075B5Q6	1,0	3,3	13,4	0,7	12,1	Immunoglobulin heavy variable 5-9-1
25	A0A075B5T6	0,0	1,0	6,4	0,0	10,7	Immunoglobulin heavy variable V10-3 (Fragment)
26	A0A075B5U6	1,0	1,8	19,1	0,0	13,9	Immunoglobulin heavy variable V1-20
27	A0A075B5T5	1,0	4,5	8,6	1,1	3,7	Immunoglobulin heavy variable V1-5
28	A0A075B5T9	1,0	0,3	2,2	0,2	2,3	Immunoglobulin heavy variable V1-9
29	P01592	1,0	6,4	29,0	2,7	13,5	Immunoglobulin J chain
30	A0A140T8M0	1,0	1,7	10,8	1,2	11,8	Immunoglobulin kappa variable 1-117 (Fragment)
31	A0A140T8M8	1,0	1,6	10,4	3,7	12,3	Immunoglobulin kappa variable 1-131 (Fragment)
32	A0A075B5K0	1,0	2,2	0,0	0,5	0,8	Immunoglobulin kappa variable 14-126 (Fragment)
33	A0A0G2JEY5	1,0	6,2	3,2	0,2	2,6	Immunoglobulin kappa variable 4-81 (Fragment)
34	A0A140T8M4	0,0	1,0	4,0	0,9	7,4	Immunoglobulin kappa variable 8-19
35	A0A140T8P7	0,0	0,0	1,0	0,0	0,0	Immunoglobulin kappa variable 8-21 (Fragment)
36	Q60963	1,0	2,8	2,5	0,2	3,8	Platelet-activating factor acetylhydrolase
37	A0A571BEV2	0,0	0,0	1,0	2,4	3,6	Predicted gene 8251
38	Q9JJ26	1,0	23,3	130,8	2,3	32,5	Pyrin
39	B2RX47	1,0	1,2	0,6	1,4	0,3	RIKEN cDNA 4930433I11 gene
40	Q8K440	1,0	0,8	0,0	2,8	0,0	ABC-type organic anion transporter ABCA8B
41	E9Q1K3	1,0	3,2	4,5	0,5	4,1	Alpha-adducin
42	B9EIX2	1,0	0,2	1,1	0,0	1,5	AW555464 protein
43	A0A0R4J1T8	1,0	0,0	77,6	0,0	13,1	Beta-alanine-activating enzyme (Fragment)
44	P13634	1,0	0,6	0,6	0,1	0,5	Carbonic anhydrase 1
45	D3Z5G7	1,0	1,4	1,0	0,6	5,4	Carboxylic ester hydrolase
46	E9QKK1	1,0	4,2	6,4	3,1	4,0	Centromere-associated protein E
47	Q6A078	1,0	1,5	14,2	0,2	0,9	Centrosomal protein of 290 kDa
48	P07310	1,0	0,2	0,1	0,0	0,1	Creatine kinase M-type
49	Q5DU00	1,0	2,0	0,1	3,8	0,0	Doublecortin domain-containing protein 2
50	P11531	1,0	3,1	7,9	0,4	6,4	Dystrophin
51	Q8BPB5	0,0	1,0	1,2	0,0	4,3	EGF-containing fibulin-like extracellular matrix protein 1
52	Q8BIE6	0,0	1,0	0,4	0,0	0,1	FERM domain-containing protein 4A

53	Q923D2	1,0	0,4	1,1	0,1	0,5	Flavin reductase (NADPH)
54	S4R257	1,0	1,6	1,6	0,2	2,0	Glyceraldehyde-3-phosphate dehydrogenase (Fragment)
55	Q3V1J8	1,0	0,1	0,3	0,6	0,5	Hyaluronan-binding protein 2
56	Q6PCQ0	0,0	1,0	0,1	0,1	0,0	IQ domain-containing protein E
57	Q6IFX2	0,0	1,0	0,8	1,4	0,0	Keratin type I cytoskeletal 42
58	A0A1B0GSR9	1,0	0,0	150,0	0,0	63,9	L-lactate dehydrogenase
59	Q9QXZ0	1,0	1,2	0,4	1,1	0,2	Microtubule-actin cross-linking factor 1 isoforms 1/2/3/4
60	E9PV66	0,0	1,0	3,4	0,9	2,9	Myosin XVIIIb
61	P70670	1,0	2,0	6,0	1,3	9,3	Nascent polypeptide-associated complex subunit alpha
62	A0A0R4J268	0,0	1,0	0,0	0,5	0,0	Oncostatin-M-specific receptor subunit beta
63	P32848	1,0	0,5	0,3	0,1	0,0	Parvalbumin alpha
64	P26450	1,0	1,9	3,2	0,0	3,0	Phosphatidylinositol 3-kinase regulatory subunit alpha
65	Q3TDN0	1,0	1,8	2,2	0,2	2,6	Protein dispatched homolog 1
66	A2ALS4	1,0	0,1	0,1	0,1	0,1	Rap1 GTPase-activating protein 1
67	F8WGW3	1,0	0,6	0,0	0,0	0,1	S1 RNA-binding domain-containing protein 1
68	Q60665	1,0	0,6	0,4	1,8	0,2	Ski-like protein
69	P08228	1,0	1,2	5,5	0,0	2,7	Superoxide dismutase [Cu-Zn]
70	K3W4L0	1,0	0,8	0,2	1,5	0,6	Unconventional myosin-XVIIIa
